# Endo-β-1,4-Xylanase GH10 from *Geobacillus stearothermophilus* CECT43: Biochemical Characterization, Insights into Its Thermal Behavior, and Synergistic Effects with a β-Xylosidase

**DOI:** 10.3390/ijms27167339

**Published:** 2026-08-17

**Authors:** Alba Carrillo-Moreno, María Gabriela Álvarez-Rodríguez, Sergio Martínez-Rodríguez, Pablo Soriano-Maldonado, Josefa María Clemente-Jiménez, Francisco Javier Las Heras-Vázquez, Lellys M. Contreras

**Affiliations:** 1 Departamento de Química y Física, Universidad de Almería, 04120 Almería, Spain; albacarrillo@ual.es (A.C.-M.); pablo.soriano@ual.es (P.S.-M.); jmclemen@ual.es (J.M.C.-J.); 2 CIAMBITAL, CeiA3, University of Almería, 04120 Almería, Spain; 3 Instituto de Biotecnología Sanitaria de Elche (IDIBE), Universidad Miguel Hernández, 03202 Elche, Spain; m.alvarezr@umh.es; 4 Departamento de Bioquímica y Biología Molecular III e Inmunología, Universidad de Granada, 18071 Granada, Spain; 5 Raw Materials, Human and Environmental Health, UGR, Associated Unit of the CSIC by the IACT-CSIC, 18100 Granada, Spain

**Keywords:** endo-β-1,4-xylanase, GH10 xylanase, xylan, kinetics, thermodynamics, synergy

## Abstract

Xylan, the principal component of hemicellulose, requires the concerted action of endo- and exo-acting xylanolytic enzymes for its complete depolymerization into fermentable sugars. This study reports the biochemical and thermodynamic characterization of a glycoside hydrolase family 10 endo-β-1,4-xylanase from the thermophilic bacterium *Geobacillus stearothermophilus* CECT43 (*Gs*XynA), cloned and heterologously expressed in *Escherichia coli* BL21(DE3) and purified to homogeneity by immobilized metal-ion affinity chromatography. The 338-residue enzyme behaved as a monomer and showed maximal activity on xylan substrates at pH 6.5 and 75 °C, retaining over 90% residual activity between pH 5 and 9 and remaining stable up to 70 °C, after 60 min of incubation. Kinetic and thermodynamic analyses of thermal inactivation monitored by enzymatic activity measurements revealed an enthalpy-driven denaturation process, while circular dichroism indicated that this irreversible unfolding is kinetically, not thermodynamically, controlled. *Gs*XynA hydrolyzed beechwood xylan mainly to xylobiose and xylotetraose, consistent with mixed endo and exo activity, and was uncompetitively inhibited by 4-hydroxybenzoic acid. Combined action with the β-xylosidase *Gs*XynB2 markedly increased D-xylose release, reaching a 53.23% hydrolysis yield and a degree of synergy of 3.79 after 24 h. These results demonstrate that *Gs*XynA is a thermostable, versatile xylanase with promising potential for lignocellulosic biomass bioconversion.

## 1. Introduction

Among renewable resources, lignocellulosic biomass (LCB) is recognized as the most abundant organic material on Earth, with an estimated annual production of approximately 2 × 10^11^ tons [1]. LCB, derived primarily from agricultural, forestry, and industrial residues, consists of three major structural polymers, namely cellulose (35–55%), hemicellulose (20–40%), and lignin (10–25%), the relative proportions of which vary depending on the plant source [2]. Hemicellulose is a branched heteropolysaccharide present in plant cell walls. Xylan is the main component of hemicellulose, which accounts for approximately one-third of the renewable organic carbon on Earth [3].

Xylan comprises a β-(1 → 4)-linked D-xylopyranose backbone decorated with L-arabinose, D-galactose, D-mannose, and organic acids [3,4,5]. Its complete degradation requires the synergistic action of primary and accessory xylanolytic enzymes [6,7,8,9,10]. Within the first group are endo-β-1,4-xylanases (EC 3.2.1.8) and β-xylosidases (EC 3.2.1.37): the former cleave internal backbone bonds to release xylooligosaccharides (XOS), which are further hydrolyzed by the latter into xylose, relieving end-product inhibition of the endoxylanases [7,9,11]. Xylanolytic enzymes are classified into glycoside hydrolase (GH) families in the CAZy database [4,12]. Endo-β-1,4-xylanases occur in nine families (GH5, GH7, GH8, GH10, GH11, GH30, GH43, GH51, and GH141), with GH10 and GH11 accounting for nearly 90% of characterized endoxylanases [3,6,9,13]. Both share a retaining mechanism, but GH10 enzymes (mostly bacterial) adopt a TIM-barrel fold with broad specificity, whereas GH11 enzymes (mostly fungal) display a β-jelly roll fold with strict specificity for unsubstituted regions, hence referred to as ‘true xylanases’ [3,8,9,14,15].

Xylanases have broad industrial applications across multiple sectors, including the biofuel and paper industries, where they enable efficient conversion of LCB and reduce the use of organochlorine compounds in pulp bleaching [2,4,7,8,15,16]. They are also widely used in the bakery, brewing and malting, pharmaceutical, and animal feed industries [16]. In particular, within the pharmaceutical and food sectors, notable advances include the production of XOS with recognized prebiotic activity [17,18] as well as the generation of xylitol, a low-glycemic sweetener suitable for diabetic consumers [19].

In recent years, bacterial xylanases have gained increasing attention due to their superior pH tolerance and thermal stability compared to their fungal counterparts. Among the diverse microbial sources of xylanolytic enzymes, several strains of the thermophilic Gram-positive bacterium *Geobacillus stearothermophilus* have emerged as particularly interesting organisms owing to their ability to produce thermostable hemicellulases [3,8,20]. One such strain is T6, which harbors a highly efficient and complete xylan-degradation system comprising 27 genes [21,22,23]. This system involves the action of two independent xylanases. An extracellular endo-β-1,4-xylanase, XT6, hydrolyzes xylan [24,25], producing short substituted XOS, that can be subsequently transported into the cells. Once in the cytoplasm, several intracellular enzymes remove side-chain substitutions, yielding short linear XOS. Complete hydrolysis of these products requires the action of exo-xylanases, as well as an intracellular xylanase known as IXT6 [22,26]. Although in *G. stearothermophilus* T-6 both the extra- and intracellular xylanases belong to the GH10 family, they exhibit distinct substrate specificities: XT6 preferentially acts on long, branched chains, whereas IXT6 displays higher affinity toward short-chain XOS. This difference has been attributed to the presence of a unique additional subdomain in XT6 [22]. In contrast to the substantial number of studies on the extracellular endo-xylanase XT6—underscoring its prominent role in the xylanolytic system of *G. stearothermophilus* T-6 [3,22,23,24,25,27,28,29,30,31,32,33]—research on IXT6 remains limited [22,26,27]. Moreover, studies on extracellular or intracellular xylanases from other strains of *G. stearothermophilus* [34,35], as well as from other *Geobacillus* species [36,37,38,39] and *Bacillus* species [40], remain limited.

The synergistic action of xylanolytic enzymes is considered essential for the efficient and complete degradation of xylan [41]. Consequently, evaluating the interactions among these enzymes has become an important strategy for improving the efficiency and reducing the cost of lignocellulosic biomass saccharification. In this context, synergistic effects have been reported between XT6, debranching enzymes, and a fixed minimum amount of xylosidase during the hydrolysis of wheat flour arabinoxylan [6]. Similarly, Anand et al. [42] described synergistic interactions between an extracellular endo-xylanase and a β-xylosidase from *G. thermodenitrificans* TSAA1 in the degradation of birchwood xylan and agro-residues.

Given the limited body of research on intracellular xylanases, it remains unclear whether they share the broad substrate specificity and thermal robustness reported for the extracellular xylanases. In this study, we address this gap by cloning, heterologously expressing, and biochemically characterizing a novel GH10 endo-xylanase, *Gs*XynA, from *G. stearothermophilus* CECT43, a thermophilic strain widely used as a reference organism for validating heat-sterilization processes. Specifically, we aimed to determine (i) the substrate range and catalytic properties of *Gs*XynA and (ii) its thermal stability and synergistic behavior with a β-xylosidase during xylan hydrolysis. We hypothesized that *Gs*XynA would display broad substrate specificity, comparable to other well-characterized GH10 xylanases, and, considering the thermophilic origin of the source strain, high thermal stability—both of which were subsequently evaluated through thermodynamic, kinetic, and biochemical analyses. Detailed characterization demonstrated its ability to hydrolyze a broad range of xylans, highlighting its catalytic versatility. Altogether, these analyses provide an in-depth characterization of *Gs*XynA and highlight its potential application in the sustainable bioconversion of lignocellulosic biomass.

## 2. Results

### 2.1. Cloning and Sequence Analysis of GsXynA

The endo-1,4-xylanase gene was sourced from *Geobacillus stearothermophilus* CECT43, a strain deposited at the Spanish Type Culture Collection (CECT) that corresponds to the type strain *G. stearothermophilus* ATCC 12980 (genome accession number GCA_001277805). A 1104 bp fragment was successfully amplified by PCR using genomic DNA of *G. stearothermophilus* CECT43 as a template; cloned into the pET-21b(+) plasmid, yielding the recombinant vector pJMC118; and propagated in *E. coli* DH5α. DNA sequencing confirmed that the insert encodes a polypeptide of 338 amino acids belonging to GH family 10. No signal peptide was identified in *Gs*XynA, indicating that it is an intracellular xylanase, a result consistent with the use of cloning primers designed based on GenBank sequence accession number D28121.1.

**Figure 1:**
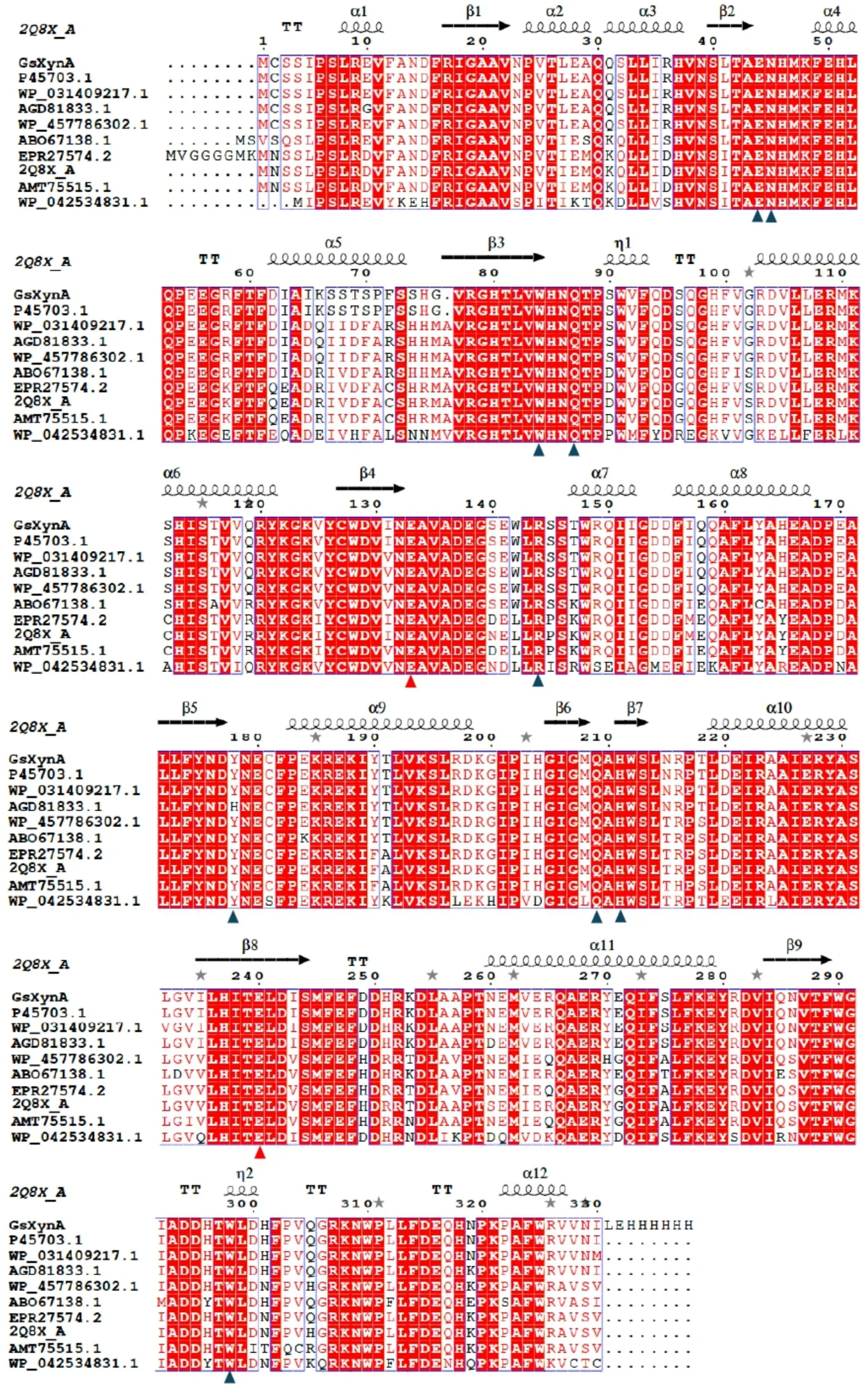
Multiple sequence alignment of the intracellular GH10 endo-1,4-β-xylanase *Gs*XynA from *Geobacillus stearothermophilus* CECT43 with representative xylanases from *Geobacillus* and *Anoxybacillus* strains. The GenBank or UniProt accession number for the aligned sequences are P45703.1 (*G. stearothermophilus* No. 21); WP_031409217.1 (multispecies *Geobacillus* spp.); AGD81833.1 (*G. stearothermophilus* strain 1A05583); WP_457786302.1 (*Geobacillus* sp. Geo 8.1); ABO67138.1 (*G. thermodenitrificans* NG80-2); EPR27574.2 (*Geobacillus* sp. WSUCF1); 2Q8X_A (*G. stearothermophilus* T6); AMT75515.1 (*Geobacillus* sp. TF16); and WP_042534831.1 (*Anoxybacillus ayderensis*). Secondary structure elements depicted above the alignment are derived from the crystal structure of *G. stearothermophilus* T6 xylanase (PDB: 2Q8X), where α-helices, 3_10_-helices, β-strands and strict β-turns are represented by squiggles, η symbols, arrows, and TT letters, respectively. Residues identical in all aligned sequences are displayed on a red background; those with conservative substitutions are enclosed in blue boxes. Substrate-binding and catalytic residues are indicated by blue and red triangles, respectively. Amino acids that do not have a single, fixed position in the 3D structure file (PDB: 2Q8X_A) used as a reference are marked with a gray star. The figure was generated using ESPript 3.0 [43].

BLASTp (version 2.17.0) analysis of the *Gs*XynA amino acid sequence showed the highest identity with the annotated endo-1,4-β-xylanase from *Bacillus stearothermophilus* 21 (P45703.1; 100%), followed by xylanases from different *Geobacillus* species (WP_031409217.1; 95.77%), the endo-1,4-beta-xylanase from *Geobacillus stearothermophilus* strain 1A05583 (AGD81833.1; 95.17%), the endo-1,4-β-xylanase from *Geobacillus* sp. Geo 8.1 (WP_457786302.1; 90.03%), the intracellular xylanase from *Geobacillus thermodenitrificans* NG80-2 (ABO67138.1; 85.20%), both the endo-1,4-β-xylanase from *Geobacillus* sp. WSUCF1 (EPR27574.2) and the intracellular xylanase from *G. stearothermophilus* T6 (2Q8X_A) (82.48%), the endo-β-1,4-xylanase from *Geobacillus* sp. TF16 (AMT75515.1; 80.97%), and the endo-1,4-β-xylanase from *Anoxybacillus ayderensis* (WP_042534831.1; 72.09%). The amino acid sequence of *Gs*XynA and those of other GH10 xylanases were aligned using the ESPript program [43] (Figure 1). Sequence alignment identified Glu133 and Glu240 as the respective acid–base catalyst and nucleophile of *Gs*XynA, showing full conservation across all analyzed GH10 endo-xylanases. Furthermore, the active site residues Glu44, Asn45, Trp84, Gln87, Arg144, Tyr178, Gln209, His211, and Trp298 are strictly conserved in *Gs*XynA. The alignment also identified Phe248 and Trp290 as the aromatic residues at subsites +2 and −2 in *Gs*XynA, in addition to Tyr178 and Trp298 at subsites +1 and −1, respectively. These residue assignments were guided by homology with the intracellular GH10 xylanase from *G. stearothermophilus* T-6, whose corresponding catalytic and active-site residues (Glu134, Glu241, Glu44, Asn45, Trp85, Gln88, Arg145, Tyr179, Gln210, His212, and Trp299) were structurally identified by Solomon et al. [22].

### 2.2. Expression and Purification of GsXynA

**Figure 2:**
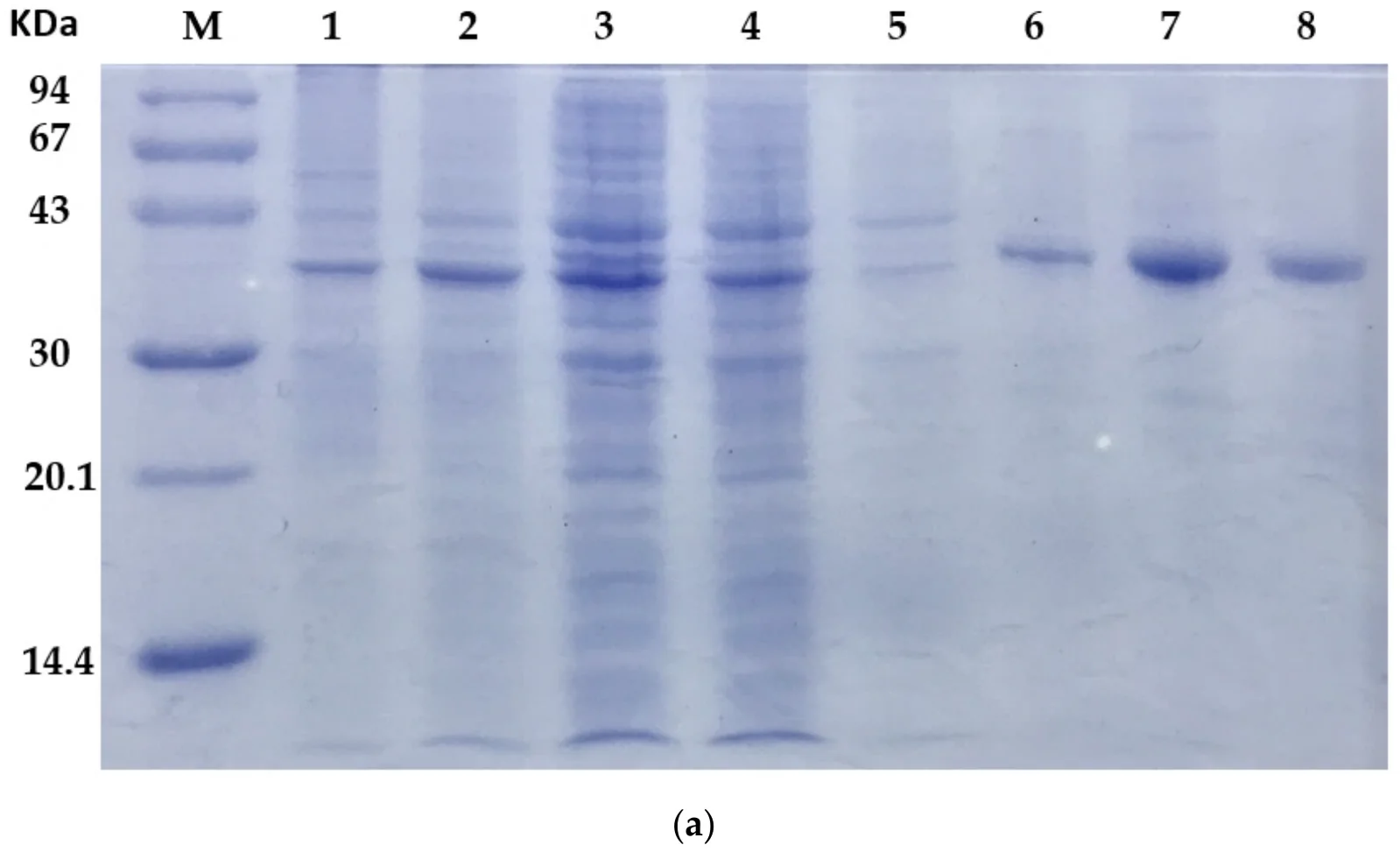

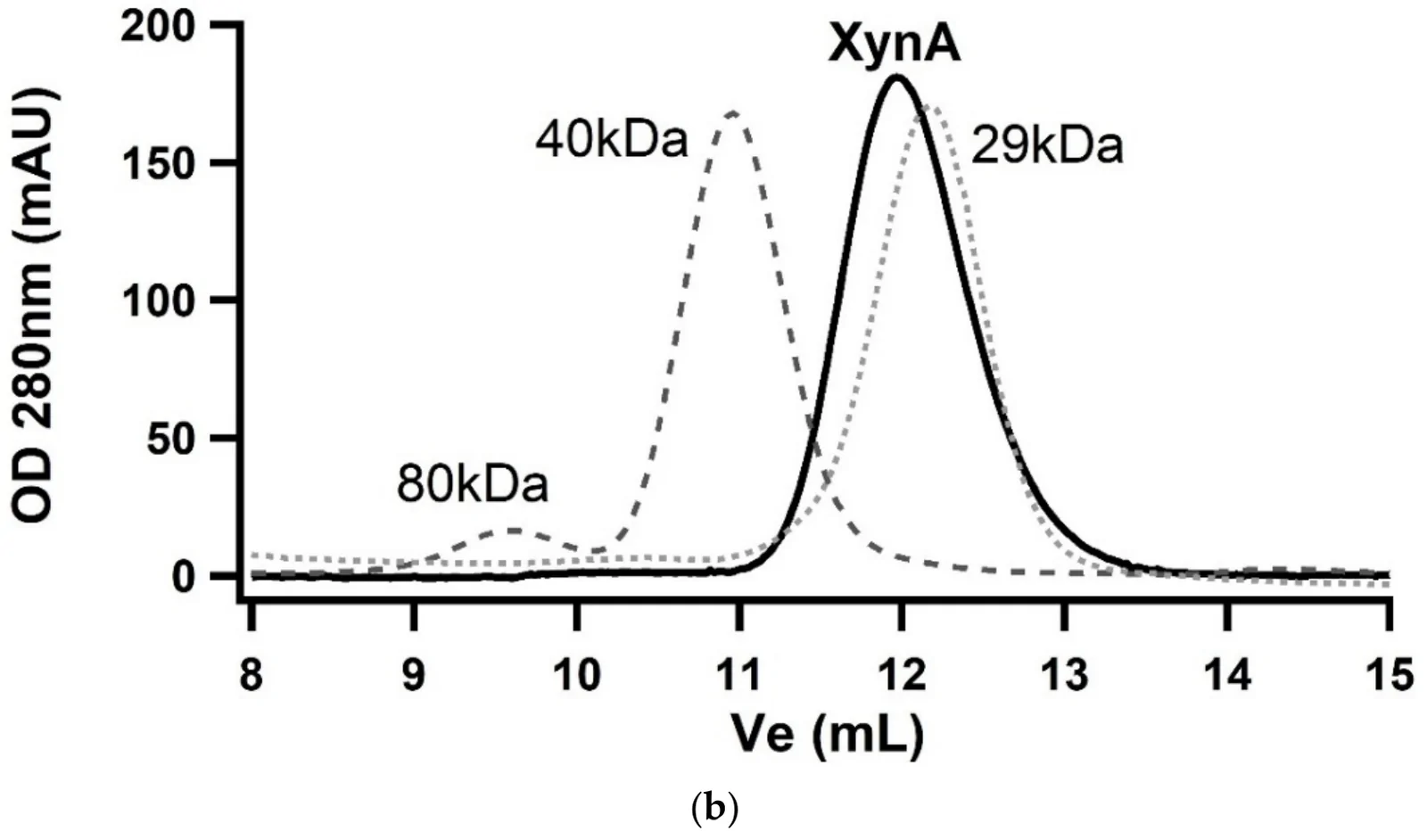
(**a**) SDS-PAGE analysis of *Gs*XynA. Lane M: low-molecular weight markers; Lane 1: non-induced cells; Lane 2: induced pellet; Lane 3: induced supernatant; Lane 4: flow-through; Lane 5: column wash; Lanes 6–8: eluted fractions 2–4. (**b**) SEC-FPLC of recombinant *Gs*XynA (solid black line) using a Superdex 75 column, with 20 mM Tris-HCl pH 8.0 150 mM NaCl as running buffer. Carbonic anhydrase (~29 kDa) and ovoalbumin (~40 and ~80 kDa) were injected as standards (dashed gray lines).

Recombinant *Gs*XynA was expressed in *E. coli* BL21 (DE3) cells and subsequently purified to electrophoretic homogeneity from the clarified cell lysate using IMAC. SDS-PAGE analysis using a 15% (*w*/*v*) gel showed a distinct band between the 43 and 30 kDa markers (Figure 2a, lanes 6–8), a molecular mass consistent with the theoretical molecular mass of *Gs*XynA (39.5 kDa) calculated using the ExPaSy ProtParam server [44]. The protein band in the induced supernatant (Figure 2a, lane 3) confirmed that *Gs*XynA was expressed in soluble form. Lane 4 showed no enzyme band, indicating that *Gs*XynA in the induced supernatant had bound to the resin. The absence of the target band in the column wash (Figure 2a, lane 5) confirmed efficient binding to the Co^2+^ chelating resin. Finally, lanes 6–8 showed a single band, with the highest protein concentration in lane 7. These results demonstrated that *Gs*XynA is largely expressed in soluble form, with no inclusion body formation.

Among the 143 PDB entries retrieved through a protein BLAST (version 2.17.0) search using *Gs*XynA as the query, 11 share 50–82% sequence identity with it. The closest homologs belong to *Geobacillus* species (*G. stearothermophilus* or *G. proteiniphilus*), followed by *Thermobacillus composti*, *Paenibacillus barcinonensis*, and two members of the genus *Caldicellulosiruptor*. The only three-dimensional structure available among the xylanases included in the sequence alignment in Figure 1 is that of *G. stearothermophilus* T-6 (PDB: 2Q8X); this crystal structure shows two IXT6 molecules in the asymmetric unit [22]. Since SDS-PAGE is performed under denaturing conditions and therefore cannot provide information on the native oligomeric state of the protein, this was instead assessed by size-exclusion chromatography under native conditions. In the case of *Gs*XynA, the elution peak at pH 8.0 using a Superdex 75 column was located between the signals from carbonic anhydrase and ovalbumin, consistent with a monomeric form in solution (Figure 2b).

### 2.3. Protein Characterization of GsXynA by Activity Measurements

Substrate specificity. The substrate specificity of *Gs*XynA was characterized by measuring its specific activity against a panel of naturally occurring substrates, namely beechwood xylan, birchwood xylan, oat xylan, and wheat arabinoxylan, as well as the synthetic substrates carboxymethylcellulose (CMC) and aryl glycosides. As shown in Table 1, *Gs*XynA displayed the highest specific activity toward xylans. Oat spelt xylan was the least efficiently hydrolyzed among the xylans tested. Across the four xylans tested, the activity of *Gs*XynA on wheat arabinoxylan was higher than that observed for beechwood or birchwood xylans. When *Gs*XynA was tested on CMC, it exhibited measurable hydrolytic activity; however, the specific activity on this substrate was considerably lower than that observed against xylans. Following the characterization and validation of *Gs*XynA on polymeric substrates such as xylan and CMC, the analysis was extended to aryl substrates, using *p*-NPXylX6 as the initial substrate given its five-unit xylose composition. *Gs*XynA showed activity on *p*-XylX6 and *p*-NPX2, whereas virtually no activity was detected with *p*-NPX, *o*-NPX, or *p*-NPG (Table 1).

**Figure 3:**
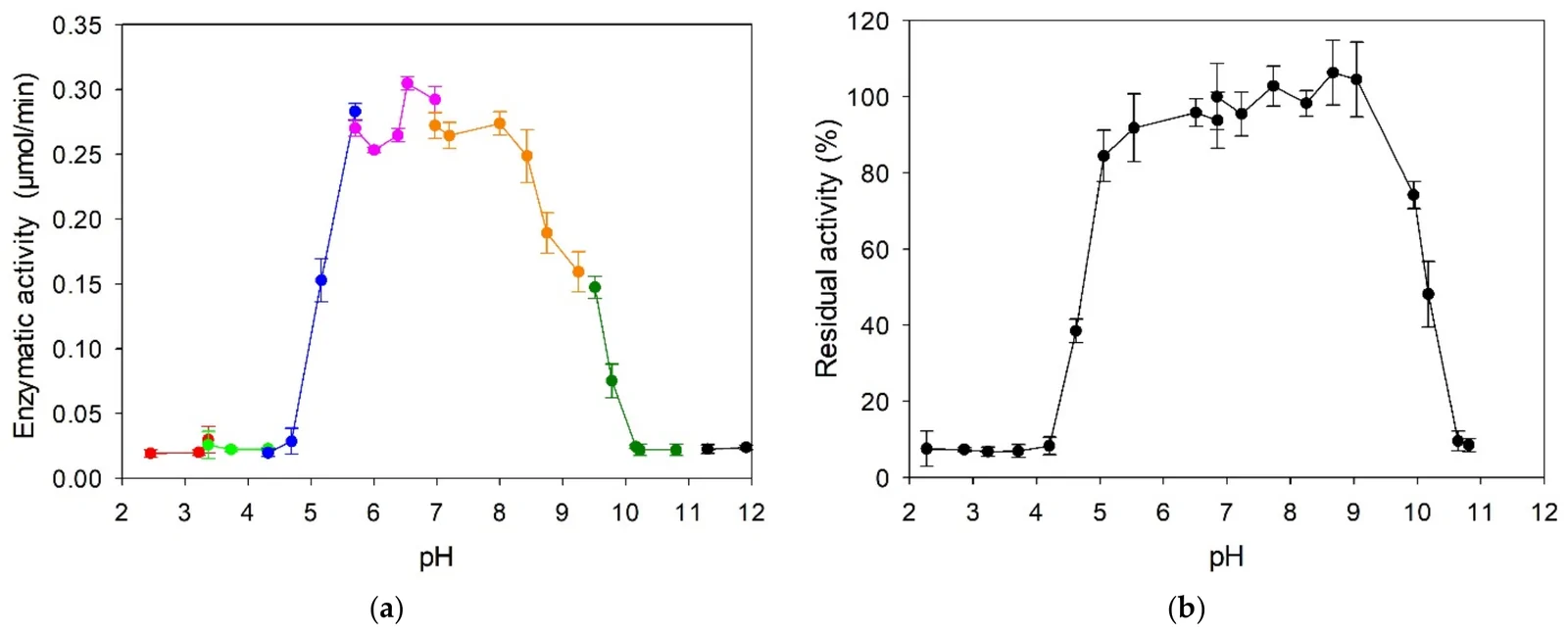

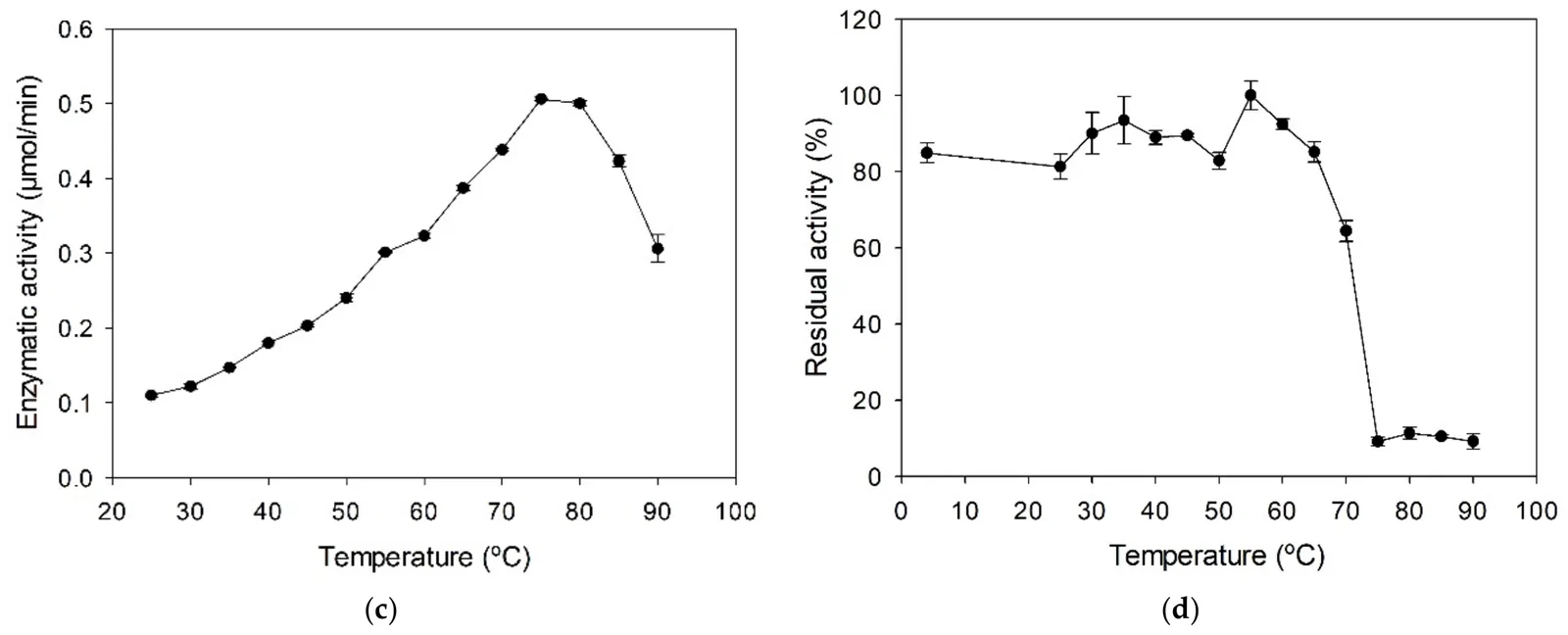
Effect of pH and temperature on enzyme activity and stability. (**a**) Effect of pH on the activity of *Gs*XynA using beechwood xylan as substrate, assessed at 55 °C across different pH values using 100 mM formate (pH 1.92–2.93, red filled circles), formic acid (pH 3.0–4.0, green filled circles), acetate (pH 4.0–5.51, blue filled circles), phosphate (pH 5.8–7.0 pink filled circles), Tris (pH 7.0–9.25, orange filled circles), carbonate (pH 9.42–10.93, dark green filled circles), and phosphate (pH 11.3–11.9, black filled circles). (**b**) Effect of pH on enzyme stability. *Gs*XynA was incubated in different pH buffers for 12 h at 4 °C before activity evaluation. (**c**) Effect of temperature on enzyme activity at pH 6.5 in 100 mM phosphate buffer. (**d**) Effect of temperature on enzyme stability. *Gs*XynA was incubated for 1 h at the indicated temperatures before activity measurement. Values shown represent mean ± standard deviations of three independent experiments.

pH and temperature profile and stability of *Gs*XynA. The optimal reaction parameters and stability profile of *Gs*XynA were assessed by determining its pH and temperature activity curves using beechwood xylan as substrate (Figure 3). The enzyme showed maximum activity at pH 6.5 and remained highly active across a broad pH range (5.5–9.5), retaining over 50% of its maximal activity at the extremes of this range (Figure 3a). To confirm that the observed pH-dependent activity profile was independent of the specific buffer system used, additional cross-validation assays were performed at the boundaries between consecutive buffer systems, using formic acid buffer at pH 4.3, and phosphate buffer at pH 5.8 and 7.0. In all cases, similar activity values were obtained with the different buffers at the same pH, confirming that the observed pH-dependent activity profile is attributable to pH itself rather than to the specific buffer (or ion) used. The pH stability of *Gs*XynA was determined by pre-incubating the enzyme at 4 °C in different buffers for 12 h (Figure 3b). As shown, *Gs*XynA exhibited remarkable stability across a broad pH range, retaining more than 90% of its residual activity at pH 5–9, while residual activity declined to 45% at pH 10.

*Gs*XynA displayed optimal activity at 75 °C (Figure 3c), retaining more than 80% of its relative activity at 65, 70, 80 and 85 °C. Thermal stability of *Gs*XynA was explored after incubation at temperatures ranging from 4 °C to 90 °C for 1 h. As shown in Figure 3d, *Gs*XynA remained stable across the 55–70 °C range, with no significant loss of performance, but exhibited a sharp drop in activity after 1 h of incubation at 75 °C. The temperature at which *Gs*XynA lost 50% of its activity (or half-inactivation temperature) was 69.97 °C, and it was very close to the value reported for XT6 (70.1 °C) [30].

### 2.4. Kinetic Parameters and Thermodynamics of Beechwood Xylan Hydrolysis

Based on the specific activities previously determined for *Gs*XynA on four xylan substrates, the three xylans that yielded the highest activities—wheat arabinoxylan, birchwood xylan, and beechwood xylan—were selected for kinetic characterization (Figure S1a–c). In addition, the aryl-glycoside substrates *p*-NPXylX6 and *p*-NPX2 were included in the analysis (Figure S1d,e). Kinetic assays were performed using 25 nM of enzyme. Although the optimal temperature for *Gs*XynA activity is 75 °C, the assays were carried out at 50 °C, since *p*-NPXylX6 is stable only up to this temperature [45]. All curves showed the typical Michaelis–Menten behavior, as expected.

The catalytic properties and kinetic parameters obtained for *Gs*XynA on all substrates tested are displayed in Table 2. *Gs*XynA exhibited comparable *K*_m_ values for the three xylans tested (3.24 ± 0.82 mg/mL for beechwood xylan, 3.36 ± 0.62 mg/mL for birchwood xylan, and 3.41 ± 0.56 mg/mL for wheat arabinoxylan), with corresponding *k*_cat_/*K*_m_ values of 221.80, 83.33, and 115.93 s^−1^·mg^−1^·mL, respectively. Interestingly, the *K*_m_ values are consistent with the range reported for extracellular and intracellular endo-xylanases from *Bacillus* and *Geobacillus* species acting on several xylans [3,24,30,35,36,40,42,46,47,48,49,50]. Given that the highest activity of *Gs*XynA on aryl β-glucosides was observed with *p*-NPX2 and *p*-NPXylX6 (Table 1), the kinetic parameters for both substrates were also determined (Table 2). Among all substrates tested—natural and artificial alike—*Gs*XynA displayed its greatest affinity for *p*-NPX2, with a *K*_m_ as low as 0.06 ± 0.01 mg/mL and a *k*_cat_/*K*_m_ of 544.50 s^−1^·mg^−1^·mL, followed by *p*-NPXylX6 (*K*_m_ = 0.56 ± 0.13 mg/mL; *k*_cat_/*K*_m_ = 44.05 s^−1^·mg^−1^·mL). Since *Gs*XynA exhibited its highest catalytic efficiency on beechwood xylan among the heteroxylans tested, the dependence of *V*_max_ on temperature was examined over the 50–75 °C range in order to obtain the activation energy (*E*_a_) of the reaction, defined as the minimum energy required for the enzyme-substrate complex to reach the transition state and proceed to product formation. The resulting Arrhenius plot of ln *V*_max_ vs. 1/T (Figure S2) yielded an activation energy (*E*_a_) of 35.33 kJ·mol^−1^ for beechwood xylan hydrolysis by *Gs*XynA.

### 2.5. Thermodynamics of Thermal Inactivation of GsXynA

**Figure 4:**
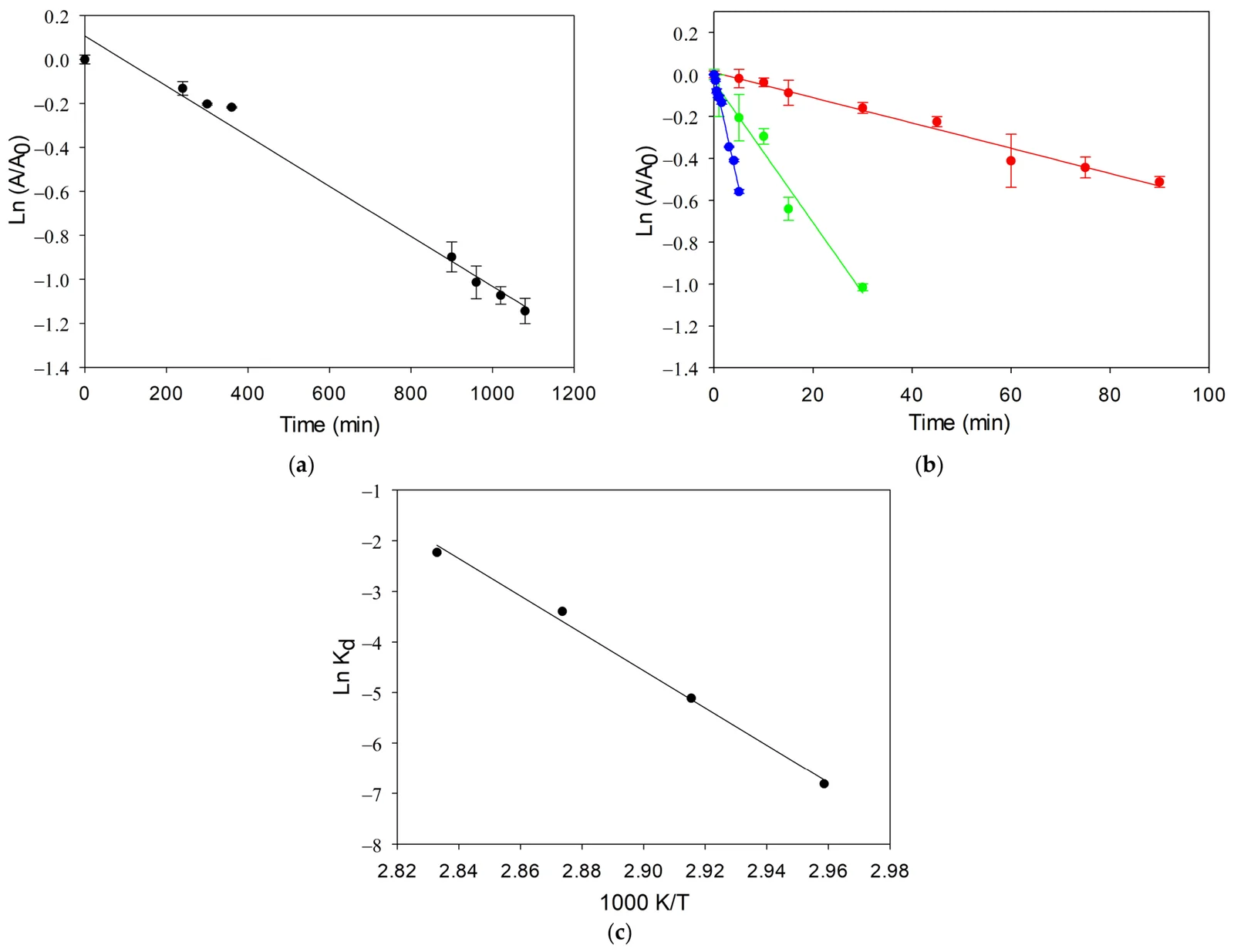
(**a**) Pseudo-first-order plot for the thermal inactivation of *Gs*XynA at 65 °C. (**b**) Pseudo-first-order plot for the thermal inactivation of *Gs*XynA at 80 °C (blue closed circles), 75 °C (green closed circles), and 70 °C (red closed circles). (**c**) Arrhenius plot for calculation of the activation energy (*E*_d_) of irreversible thermal denaturation of *Gs*XynA.

To further characterize the thermostability of *Gs*XynA, its thermal inactivation was analyzed from a kinetic and thermodynamic perspective. Figure 4a,b show the pseudo-first-order plots of ln(A/A_0_) versus heating time for *Gs*XynA inactivation at four temperatures. In Figure 4a, the decline in ln(A/A_0_) at 65 °C is gradual, with the enzyme retaining roughly 50% of its initial activity after approximately 10 h of incubation. In Figure 4b, at 70 °C (red closed circles), inactivation proceeds markedly faster, with the enzyme requiring at least 2 h to lose 50% of its activity, still reflecting a moderate level of stability. At 75 and 80 °C (green and blue closed circles, respectively), the curves drop steeply within the first few minutes of heating, reflecting a much more rapid and pronounced loss of enzymatic activity. Since, all the curves were linear, with R^2^ values greater than 0.97, the apparent inactivation rate constant (*K*_d_) at each temperature could be estimated from the slope of the plot. As shown in Table 3, *K*_d_ grows steadily with temperature, while *t*_1/2_ and the *D*-value decrease as the temperature increases. At 70 °C, *Gs*XynA exhibited a *t*_1_/_2_ of 115 min and a *D*-value of 383.8 min; these values decreased to 20.6 min and 68.5 min, respectively, at 75 °C.

In addition to these kinetic parameters, the thermodynamic parameters governing the irreversible thermal inactivation of *Gs*XynA were also determined. From the Arrhenius plot (Figure 4c), the activation energy of denaturation (*E*_d_) of *Gs*XynA, along with all the thermodynamic parameters governing its irreversible thermal inactivation, was determined. In this context, *E*_d_ represents the minimum amount of energy that enzyme molecules must acquire before unfolding can take place, and for *Gs*XynA this value was estimated at 307.08 kJ·mol^−1^. The thermodynamic parameters for thermal inactivation of *Gs*XynA, calculated over the temperature range of 65–80 °C, are depicted in Table 3. The enthalpy of denaturation (ΔH*), which reflects the number of bonds broken during enzyme inactivation, was derived directly from *E*_d_ and showed a slight decrease with increasing temperature, ranging from 304.28 kJ·mol^−1^ at 65 °C to 304.15 kJ·mol^−1^ at 80 °C. ΔG* values for *Gs*XynA declined progressively with increasing temperature, from 113.78 kJ·mol^−1^ at 65 °C to 105.51 kJ·mol^−1^ at 80 °C. Likewise, the values of ΔS* for *Gs*XynA over the temperature range of 65–80 °C were positive.

### 2.6. Secondary Structure and Thermal Denaturation of GsXynA by Circular Dichroism

**Figure 5:**
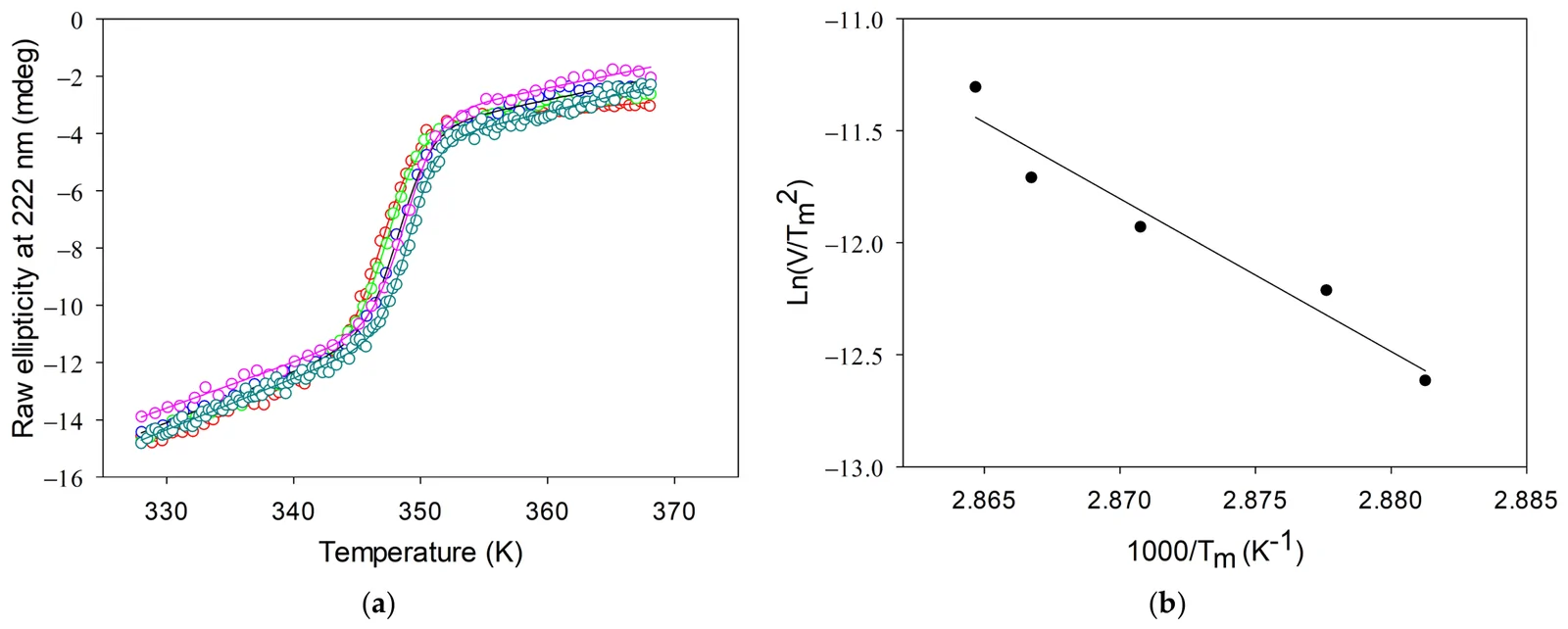
(**a**) Thermal denaturations of *Gs*XynA monitored by circular dichroism (CD) at different scan rates: 0.4 °C/min (red), 0.6 °C/min (green), 0.8 °C/min (blue), 1.0 °C/min (magenta), and 1.5 °C/min (cyan). Symbols represent experimental data points, and solid lines represent the fit of the data to the two-state-transition model. (**b**) Plot of Ln (V/*T*_m_^2^) versus 1000/*T*_m_. The goodness of the fit was estimated from the value of R^2^, which was greater than 0.945.

Circular dichroism (CD) spectroscopy in the far-UV region (190–250 nm) is a widely used technique for the rapid determination of the secondary structure composition of proteins in solution [51,52,53,54]. In addition, CD spectroscopy can be used to monitor thermally induced conformational changes, providing information on the unfolding behavior of a protein as a function of temperature. As shown in Figure S3a, the far-UV CD spectrum of *Gs*XynA at pH 6.5 displayed a positive band around 192 nm and a broad negative minimum between 208 and 222 nm, a spectral profile characteristic of protein with mixed α/β secondary structure content, consistent with the canonical (α/β)_8_ TIM-barrel fold of GH10 family xylanases [9,16]. Deconvolution of the CD spectrum using CDNN software (version 2.1) [55] yielded an estimated secondary structure composition of 40.9% α-helix, 4.8% antiparallel β-sheet, 7.8% parallel β-sheet, 16.2% β-turns, and 30.3% random coil. In contrast, the CD spectrum of the heat-denatured protein (short-dashed line), obtained after a thermal scan from 55 to 95 °C at a temperature ramp of 1.0 °C·min^−1^, no longer displays this characteristic double-minimum profile. In addition, the overall signal amplitude is markedly reduced. During this thermal scan, the detector high-tension (HT) voltage—which reflects sample turbidity—was recorded together with the ellipticity signal at 222 nm, and showed a marked increase upon denaturation (Figure S3b). The associated thermal unfolding curve, displayed as the curve with magenta symbols in Figure 5a, yielded an apparent *T*_m_ of 348.83 K.

Following the heating scan, protein precipitation was observed, which precluded the thermodynamic characterization of the denaturation process using conventional equilibrium approaches. For this reason, the model proposed by Sánchez-Ruiz et al. [56] for irreversible thermal transitions was applied. To explore the kinetics of this transition, ellipticity at 222 nm was monitored under different temperature ramps (Figure 5a), revealing a scan-rate dependence of the apparent *T*_m_, which rose from 347.07 K at 0.4 °C·min^−1^ to 349.08 K at 1.5 °C·min^−1^. The scan-rate dependence of the *T*_m_ observed for *Gs*XynA indicates that its thermal denaturation is kinetically controlled, rather than a true equilibrium process, as previously observed for other xylanolytic enzymes, likewise β-xylosidase from *G. stearothermophilus* CECT43 XynB2 [54]. Analysis of the irreversible CD thermal transitions using a two-state kinetic model yielded a linear relationship between ln(V/*T*_m_^2^) and 1/*T*_m_ (Figure 5b), with the slope corresponding to −*E*_d_/R. The resulting activation energy of denaturation was *E*_d_ = 567.08 kJ·mol^−1^, notably higher than that the value derived from thermal inactivation kinetics measured by enzymatic assays (*E*_d_ = 307.08 kJ·mol^−1^).

### 2.7. Effect of Phenolic Compounds on GsXynA Activity

To evaluate the possible inhibitory effect of lignocellulose-derived phenolic compounds on *Gs*XynA, the effect of three different concentrations of 4-hydroxybenzaldehyde, vanillin, 4-hydroxyacetophenone, acetosyringone, 4-hydroxybenzoic acid, and *p*-coumaric acid was assessed on the hydrolysis of beechwood xylan (Figure S4a). Marked differences were observed among the six compounds tested: 4-hydroxybenzaldehyde and vanillin had no discernible effect on enzyme activity at any of the concentrations assayed, whereas acetosyringone and 4-hydroxyacetophenone caused a moderate, concentration-dependent decrease in activity which became noticeable only at the highest concentration tested (5 mg/mL), reaching approximately 20% inhibition in the case of acetosyringone. By contrast, 4-hydroxybenzoic acid and *p*-coumaric acid emerged as the most potent inhibitors, reducing *Gs*XynA activity by approximately 70% and 60%, respectively, at 5 mg/mL. Since all compounds were dissolved in DMSO prior to their addition to the reaction mixture, the effect of DMSO alone on *Gs*XynA activity was also assessed. DMSO was tested at 5% (*v*/*v*), the highest concentration used, corresponding to the inhibitor concentration of 5 mg/mL. As shown in Figure S4b, enzyme activity in the presence of DMSO was virtually identical to that of *Gs*XynA alone, confirming that the solvent had no effect on enzyme activity under the assay conditions. This result indicates that the inhibitory effects described above are attributable to the phenolic compounds themselves, rather than to the solvent in which they were dissolved. Interestingly, the inhibition exerted by 4-hydroxybenzoic acid on *Gs*XynA was found to be reversible (Figure S4c).

**Figure 6:**
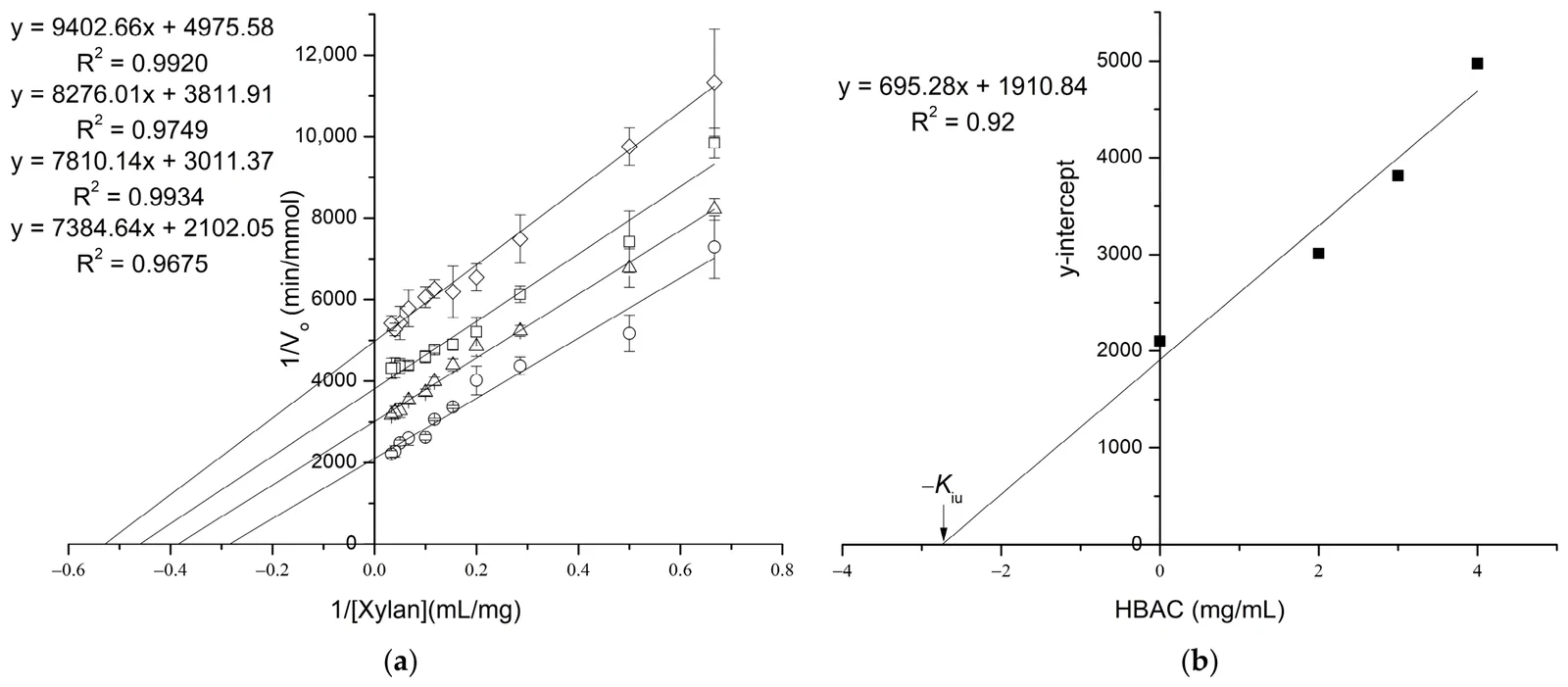
Uncompetitive inhibition of *Gs*XynA by 4-hydroxybenzoic acid. (**a**) Lineweaver-Burk plots in the absence (circles) and presence of 4HBAC: 2.5 mg/mL (triangles), 3 mg/mL (squares), and 4 mg/mL (diamonds). The goodness of the fit was estimated from the R^2^ value, which was greater than 0.9675. (**b**) Secondary plot of *y*-intercept in Lineweaver-Burk plot versus 4HBAC concentration. Values shown represent the mean ± standard deviation of three independent experiments.

Among the phenolic compounds screened, 4-hydroxybenzoic acid (4HBAC) showed the strongest inhibitory effect and was therefore selected for further characterization of its inhibition type. For this purpose, reactions were performed at different substrate concentrations in the presence of three concentrations of 4HBAC. The Michaelis-Menten kinetic parameters of *Gs*XynA differed as the concentration of inhibitor increased (Figure S4d). The corresponding Lineweaver-Burk plot (1/V_0_ vs. 1/[Xylan]) (Figure 6a) yielded a series of parallel lines. As shown in Table 4, both *K*_m_ and *V*_max_ at the three concentrations tested were lower than those of the control, and the *K*_m_/*V*_max_ ratio was close across inhibitor concentrations. A secondary replot of the y-intercept from the Lineweaver-Burk plots against 4HBAC concentration (Figure 6b) was linear, and extrapolation to the x-axis yielded an uncompetitive inhibition constant (*K*_iu_) of 2.75 mg/mL.

### 2.8. Hydrolysis Products and Synergistic Degradation of Beechwood Xylan by GsXynA and GsXynB2

**Figure 7:**
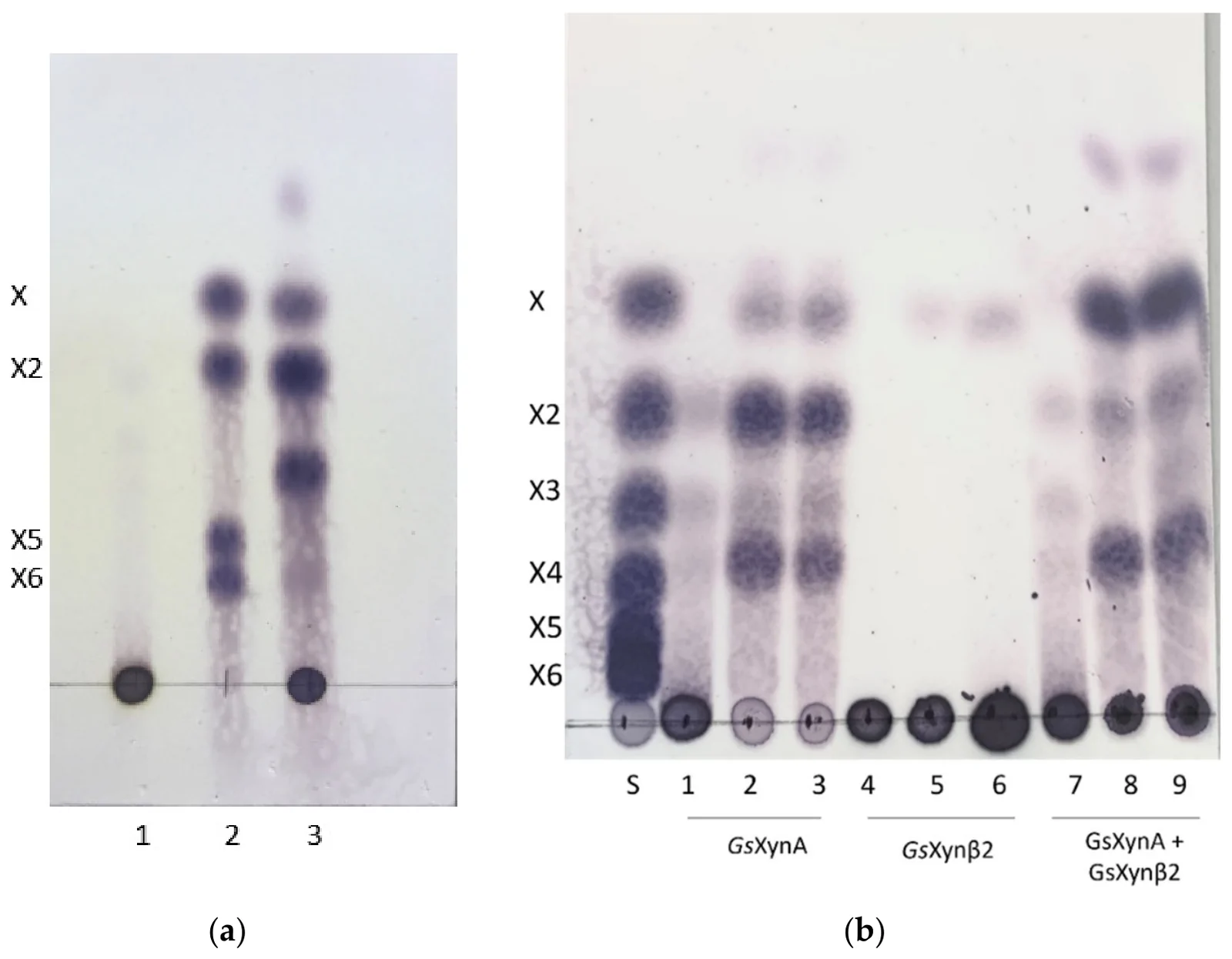
(**a**) TLC analysis of the xylooligosaccharides released from the hydrolysis of beechwood xylan by *Gs*XynA at 70 °C. Lane 1, time zero; Lane 2, standards (xylose (X), xylobiose (X2), xylopentaose (X5), and xylohexaose (X6)); Lane 3, products obtained after 12 h of incubation. (**b**) TLC analysis of the XOS released from beechwood xylan upon hydrolysis by *Gs*XynA and *Gs*XynB2, alone or in combination. Lane S, standards (xylose (X), xylobiose (X2), xylotriose (X3), xylotetraose (X4), xylopentaose (X5), and xylohexaose (X6)); Lanes 1–3, products obtained after 0, 4, and 24 h of hydrolysis by *Gs*XynA; Lanes 4–6, products obtained after the same incubation times by *Gs*XynB2; Lanes 7–9, products obtained after the same incubation times by the combined action of both enzymes.

The action of *Gs*XynA on beechwood xylan was examined after 12 h of reaction time by TLC analysis (Figure 7a). The final products consisted primarily of xylobiose and a prominent band of XOS with 3 or 4 xylose units, migrating between the X2 and X5/X6 standards, along with a small amount of xylose. Notably, this hydrolysis pattern was already established shortly after the reaction started and remained essentially unchanged across the three time points examined (4, 12, and 24 h), with xylose, xylobiose, and xylotetraose as the main products throughout (Figure 7a,b).

Building on this characterization, the enzymatic synergism between *Gs*XynA and *Gs*XynB2 [54] during the degradation of beechwood xylan was investigated by comparing three systems: *Gs*XynA alone, *Gs*XynB2 alone, and the simultaneous action of both enzymes. The TLC analysis of hydrolyzed products by *Gs*XynA alone showed the same pattern at all three reaction times tested (Figure 7b, lanes 1–3), with xylobiose (X2) and xylotetraose (X4) as the predominant products, and only a faint xylose (X) spot. In contrast, *Gs*XynB2 alone produced only a very faint xylose spot and no detectable XOS (Figure 7b, lanes 4–6). When both enzymes acted simultaneously, a markedly more intense xylose spot was observed, together with a visible reduction in the accumulation of XOS compared to *Gs*XynA alone (Figure 7b, lanes 7–9).

Furthermore, the capability of *Gs*XynA and *Gs*XynB2 alone or in combination to hydrolyze beechwood xylan is shown in Table 5. The D-xylose produced by both enzymes acting together was higher than that produced by *Gs*XynA or *Gs*XynB2 alone. At 4 h of reaction, the combined action of *Gs*XynA and *Gs*XynB2 released 3.12 mg/mL of D-xylose, corresponding to a yield of 39.06%, whereas at 24 h, the combined enzymatic system released 4.25 mg/mL of D-xylose (53.23% yield). This synergistic effect was reflected in degrees of synergy of 4.28 and 3.79 at 4 and 24 h, respectively.

## 3. Discussion

### 3.1. Sequence Conservation, Active-Site Architecture, and Oligomeric Behavior of GsXynA

*Geobacillus stearothermophilus* is a thermophilic bacterium capable of growing at high temperatures, making it a valuable source for discovering thermostable enzymes with potential biotechnological applications [8,20]. In this study, we identified a *xyna* gene in *G. stearothermophilus* CECT43 through genome analysis. The gene was amplified by PCR, enabling the construction of the recombinant plasmid pJMC118. The resulting protein, *Gs*XynA, is a 338-amino acid polypeptide lacking a signal peptide, indicating that it is an intracellular enzyme. Consistent with this classification, based on the multiple alignment of the amino acid sequence of the *Gs*XynA, we found identities ranging from 80 to 100% with endo-xylanases from several strains of *Geobacillus stearothermophilus* and other *Geobacillus* species [22,34,35,36,38]. Similar to other endo-xylanases, the active site residues in *Gs*XynA are strictly conserved and in agreement with those previously described for IXT6, the first intracellular GH10 endo-xylanase to be characterized [22]. Taken together, these findings suggest that *Gs*XynA shares the substrate recognition and catalytic mechanisms characteristic of other GH10 endoxylanases, reflecting strong evolutionary pressure to maintain both the structural integrity and catalytic function of intracellular GH10 xylanases.

This conservation at the sequence and active-site level is further reflected in the behavior of *Gs*XynA during recombinant production and structural characterization, which likewise mirrors that of related intracellular GH10 endo-xylanases. The soluble expression of recombinant intracellular GH10 xylanases has been previously reported. In fact, the expression, purification, crystallization, and X-ray analysis of IXT6, an intracellular GH10 endo-xylanase with 82.48% sequence identity to *Gs*XynA, identified it as a monomer in solution [22,26]. Furthermore, XynA2 from *G. thermodenitrificans* NG80-2 and GsXynTF16, with 85.20% and 80.97% sequence identity to *Gs*XynA, respectively, were also purified in soluble form by nickel ion affinity chromatography [36,46]. Finally, the intracellular endoxylanase from *Anoxybacillus ayderensis*, with 72.09% sequence identity to *Gs*XynA, was also purified in soluble form by heat shock treatment followed by two different chromatography steps [57].

Despite their biological importance, intracellular bacterial xylanases remain functionally and structurally underexplored, with only a limited number resolved to date. Regarding the crystal structure of *G. stearothermophilus* T-6 (PDB: 2Q8X), the crystal packing showing two IXT6 molecules in the asymmetric unit is not biologically relevant, as the enzyme behaves as a monomer in solution, as determined by size exclusion chromatography [22]. Other thermophilic and mesophilic intracellular xylanases with known three-dimensional structures, such as those from *Thermobacillus composti* and *Paenibacillus barcinonensis*, are likewise monomeric in solution [58,59], consistent with the monomeric behavior observed for *Gs*XynA.

### 3.2. Biochemical Characterization of GsXynA Activity: A Comparative Perspective

The substrate preferences and pH/temperature behavior of *Gs*XynA was analyzed in relation to previously characterized xylanases from *Geobacillus* and other genera. Previous studies have indicated that the extracellular and intracellular xylanases of *G. stearothermophilus* T6 show significant differences in substrate specificity, as IXT6 preferentially hydrolyzes short XOS [22,27]. To the best of our knowledge, a pioneering study reported enzymatic activity on oat spelt xylan for an enzyme sharing 100% sequence identity with *Gs*XynA [34]. Our results confirm this finding, as *Gs*XynA is capable of hydrolyzing oat spelt xylan, albeit with lower efficiency than xylans from other sources. This lower activity toward oat spelt xylan is consistent with observations reported for the extracellular GH10 endo-xylanase from *Thermotoga naphthophila* RKU-10T [60] and Xyn10A from *Penicillium oxalicum* GZ-2 [61]. The higher activity on wheat arabinoxylan suggests a preference for arabinose-substituted xylans, a preference that has also been reported for the intracellular GH10 endo-xylanase from *Thermobacillus composti* [58] and the extracellular GH10 endo-xylanase from *Talaromyces amestolkiae* [15]. The absence of cellulase activity is a key requirement for xylanases intended for use in the paper industry, and the considerably lower specific activity of *Gs*XynA on CMC compared to xylans is in agreement with what has been reported for other fungal and bacterial xylanases [15,60]. The ability of *Gs*XynA to hydrolyze *p*-NPXylX6, an aryl substrate composed of five xylose units, is consistent with the study by Zolonitsky et al. [27] on the affinity of non-catalytic mutants of the extracellular and intracellular xylanases of *G. stearothermophilus* T6 for distinct XOS, which revealed that IXT6 possesses only five binding subsites. Taken together, these results confirm that *Gs*XynA is a versatile glycoside hydrolase capable of degrading a wide variety of natural plant cell wall polysaccharides and artificial substrates. This is consistent with its endo-xylanase character and the known versatility of GH10 endo-xylanases, which can hydrolyze β-1,4 linkages between xylopyranose residues, including those located in the vicinity of side chain substitutions, and even certain cellulosic substrates.

Concerning the pH and temperature profile, the broad activity range and retention of over 50% of maximal activity at the extremes of the tested pH range indicate robust alkaline tolerance. Like *Gs*XynA, many bacterial GH10 endo-xylanases—both intracellular and extracellular—from the genera *Bacillus* and *Geobacillus* act optimally at neutral to alkaline pH [21,24,30,32,35,36,37,38,40,42,46,47]. Such pH stability is a common trait among bacterial GH10 endo-xylanases, as similarly reported for *G. stearothermophilus* strain 1A05583, *Bacillus* sp. HJ12, and *Bacillus safensis* [35,40,47]. In addition to this alkaline tolerance, *Gs*XynA displayed a broad thermal activity profile, remaining highly active over a 20 °C span around its optimum. The high optimal temperature is consistent with reports of elevated xylanase activity at high temperatures in several *Bacillus* and *Geobacillus* species [8,16], even though xylanases obtained from bacteria and actinomycetes typically show optimal activity within a lower range of 35–60 °C [16]. Indeed, the high-temperature range observed for *Gs*XynA closely matches the optimal temperatures reported for thermophilic bacterial xylanases, which generally fall between 55 °C and 75 °C [3,21,30,35,36,37,38,42,46,47], with the exception of xylanases from *Thermotoga* species [60]. Regarding thermal stability, the sharp decline in *Gs*XynA activity observed after 1 h of incubation at 75 °C closely matches the thermal stability behavior reported by Zhang et al. [30] for the *G. stearothermophilus* T6 extracellular endo-xylanase, which retained stable activity between 40 and 70 °C before declining markedly above 72 °C. Accordingly, the half-inactivation temperature for *Gs*XynA (69.97 °C) was very close to the value reported for XT6 (70.1 °C), even though the latter was determined after only 20 min of heat treatment, five times shorter than the 1 h incubation used in the present study. As for the thermostability of other xylanases, Wu et al. [37] reported that the xylanase from *Geobacillus* sp. MT-1, which shares 82.5% identity with *Gs*XynA, completely lost its activity after only 10 min of incubation at 75 °C. This marked contrast with the stability observed for *Gs*XynA supports the idea that the latter possesses greater thermostability. Furthermore, the broad pH stability, thermostability, and melting temperature of *Gs*XynA demonstrate its potential usefulness in the conversion of LCB.

### 3.3. Kinetic Behavior of GsXynA on Natural and Aryl Substrates in a Comparative Context

The use of chromogenic aryl-substrates alongside natural xylans in this study was motivated by earlier reports on intracellular GH10 xylanases, such as IXT6 from *G. stearothermophilus* T-6, which have been described as preferentially active on XOS rather than polymeric xylan [22]. Although the hydrolytic activity of IXT6 on polymeric xylan has not been directly reported, its catalytically inactive mutant (E134Q) has been shown to bind beechwood and birchwood xylan [27], suggesting that IXT6 may act on these substrates. *p-*NPXylX6 was used to assess endo-acting activity, as its terminal *p*-nitrophenyl group is released as a non-blocked chromogenic oligosaccharide upon endoxylanase cleavage and subsequently hydrolyzed by the ancillary β-xylosidase to release 4-nitrophenol [45]. In turn, *p*-NPX2 was included to evaluate potential exo-acting activity and, consequently, affinity for short XOS. Testing *Gs*XynA against both polymeric xylans and these defined substrates therefore allowed us to assess whether its substrate specificity followed the XOS-restricted pattern reported for IXT6.

The kinetic parameters obtained for *Gs*XynA provide a basis for evaluating its catalytic performance relative to other GH10 endo-xylanases. As stated in results, *Gs*XynA exhibits comparable affinity for all three xylans tested, a finding consistent with the range reported for extracellular and intracellular endoxylanases from *Bacillus* and *Geobacillus* species. The extracellular endo-xylanase from *Geobacillus stearothermophilus* T6 shows a *K*_m_ of 1.63 mg/mL on oat spelt xylan [24] or 0.8 mg/mL on beechwood and birchwood xylan [3,30]. By comparison, *G. thermodenitrificans* strains TSAA1 and NG80-2 display values ranging from 0.625 to 2.85 mg/mL [42,48] and 1.54 mg/mL [36], respectively, while *G. thermoleovorans* shows *K*_m_ values of 2.6 mg/mL for the wild-type and 2.1 mg/mL for the recombinant form [49]. *Bacillus safensis*, in turn, has a *K*_m_ of 1.96 mg/mL [47]. These latter values, for TSAA1, NG80-2, *G. thermoleovorans*, and *B. safensis*, were obtained using beechwood, birchwood, or oat spelt xylan. A comparable pattern emerges when endoxylanases with the highest identity to *Gs*XynA are considered. The xylanases from *G. stearothermophilus* strains 1A05583 and 21, which exhibit 95.2% and 100% identity to *Gs*XynA, respectively, have *K*_m_ values of 1.66 mg/mL on beechwood xylan and 3.8 mg/mL on oat spelt xylan [35,50]. Meanwhile, the intracellular xylanase from *G. thermodenitrificans* strain NG80-2 exhibited a *K*_m_ of 0.27 mg/mL on beechwood xylan [36]. In the case of xylanases from *Geobacillus* sp., strains MT-1 and WSUCF1 (82.48% identity to *Gs*XynA) showed *K*_m_ values of 1.579 mg/mL on oat spelt xylan and 1.57 mg/mL on birchwood xylan, respectively [37,38], while strain TF16 (80.97% identity to *Gs*XynA) showed a *K*_0.5_ of 1.75 mg/mL on xylan of unidentified origin [46]. Finally, the mesophilic intracellular xylanase from *Bacillus* sp. strain HJ2, with 60.3% identity to *Gs*XynA, showed a *K*_m_ of 0.5 mg/mL on birchwood xylan [40].

It is known that bacterial xylanases belonging to GH family 10 are able to hydrolyze the aryl β-glucosides of xylobiose and xylotriose at the aglyconic bond [8,16]. The greatest affinity of *Gs*XynA for *p*-NPX2 indicates a strong preference for short XOS. This pattern is consistent with reports for other bacterial intracellular GH10 xylanases, such as IXT6 from *G. stearothermophilus* T6 [22], although in the case of *Gs*XynA this preference does not appear to be absolute, as the enzyme also efficiently degrades xylan from grasses and woods. A comparable result has been reported for the XynSOS enzyme from *T. amestolkiae*, which likewise showed its highest affinity and catalytic efficiency with *p*-NPX2 as the substrate [15].

The catalytic efficiency of *Gs*XynA on the studied xylans falls within the same range reported for the endoxylanases of *Geobacillus stearothermophilus* strain 1A05583 [35] and *Geobacillus thermoleovorans* [49]. However, it is lower than the values reported for the extracellular xylanase of *Geobacillus stearothermophilus* T6—1178 s^−1^·mg^−1^·mL for beechwood xylan and 472 and 1303 s^−1^·mg^−1^·mL for birchwood xylan [3,30]—and for the intracellular xylanase of *Geobacillus thermodenitrificans* strain NG80-2 [36]. Taken together, these results indicate that the catalytic efficiency of *Gs*XynA is comparable to that of other *Geobacillus* endoxylanases, although lower than the highest values reported to date for the genus.

The activation energy barrier (*E*_a_) of 35.33 kJ·mol^−1^ obtained for beechwood xylan hydrolysis by *Gs*XynA can be further contextualized by comparison with other thermostable xylanases. No activation energy values for GH10 xylanases from the genus *Geobacillus* could be identified in the available literature; the *E*_a_ value obtained for *Gs*XynA was therefore compared with those reported for other thermostable bacterial xylanases. In this regard, the *E*_a_ value of *Gs*XynA is in agreement with values described for xylanases from *Bacillus safensis* (39.8 kJ·mol^−1^) [47], *B. halodurans* TSEV1 (30.51 kJ·mol^−1^ for birchwood xylan) [62], and *Bacillus methylotrophicus* CSB40 (29.39 kJ·mol^−1^ for beechwood xylan) [63].

### 3.4. Kinetic and Thermodynamic Basis of GsXynA Thermal Inactivation

Understanding the molecular basis of protein stability is essential for the characterization of thermophilic enzymes and for evaluating their potential applications in industrial biocatalysis. Enzyme thermostability is typically assessed through kinetic and thermodynamic analysis of thermal denaturation. Irreversible enzyme denaturation is generally described as a first-order deactivation process, the kinetics of which are commonly characterized in terms of deactivation rate constant (*K*_d_), half-life and *D*-value, while the corresponding thermodynamic parameters—enthalpy, entropy, Gibbs free energy, and activation energy of denaturation—provide insight into the energetics of the transition between the folded and unfolded states.

The gradual decline observed at 65 °C indicates that *Gs*XynA is highly thermostable at this temperature, whereas the markedly faster inactivation at 70 °C reflects a more moderate level of stability. The steep drop observed at 75 and 80 °C, in turn, reflects a much more rapid and pronounced loss of enzymatic activity. Few studies in the literature have examined the thermostability of endo-xylanase activity in a manner similar to the present work. For instance, a GH11 family xylanase from *Geobacillus stearothermophilus* No. 236 exhibited a *t*_1_/_2_ of only 4 min at 70 °C [64], considerably lower than the 115 min observed for *Gs*XynA at the same temperature. Similarly, lower half-life values at 70 °C were also reported for GH10 endo-xylanases from *Geobacillus thermodenitrificans* TSAA1 (*t*_1_/_2_ = 40 min) [42], *Bacillus safensis* (*t*_1_/_2_ = 3 min) [47], and *Bacillus halodurans* TSEV1 (*t*_1_/_2_ = 58 min) [62]. Although the half-life of inactivation of *Gs*XynA at 75 °C was 4.5 times greater than that of the wild-type endo-xylanase from *Geobacillus stearothermophilus* T6, it is more thermolabile than the XT6 mutant generated by 13 amino acid substitutions [30]. These results suggest that *Gs*XynA would be an excellent platform for obtaining mutants with improved thermostability.

Thermal inactivation of enzymes is generally described as a two-step process involving three states: the native enzyme (N), the unfolded enzyme (U), and the inactivated enzyme (I) [62]. When the enzyme is heated, the unfolded enzyme can still refold into its native conformation upon cooling, as long as the energy it absorbs stays below a critical threshold, the activation energy of denaturation (*E*_d_). Once the input energy surpasses this barrier, unfolding becomes irreversible, and the inactivated enzyme is formed and is unable to refold to its original state [65], with a structure that can no longer be restored. This transition is also accompanied by the disruption of covalent and non-covalent interactions, along with an increase in enthalpy (ΔH*) and entropy (ΔS*) [60]. *E*_d_ represents the minimum amount of energy that enzyme molecules must acquire before unfolding can take place. The *E*_d_ value obtained for *Gs*XynA (307.08 kJ·mol^−1^) is higher than the value reported for an endo-xylanase from *Bacillus safensis* (195.4 kJ·mol^−1^) [47], similar to that of the endo-xylanase from *Bacillus halodurans* TSEV1 (254.5 kJ·mol^−1^) [62], and lower than the values reported for the GH10 and GH11 endo-xylanases from *Thermotoga naphthophila* RKU-10T (516.3 kJ·mol^−1^) [60] and *Bacillus* sp. strain 41M-1 (518 kJ·mol^−1^) [66], respectively. The high value of *E*_d_ suggests that *Gs*XynA exhibits considerable thermostability and is highly compact. Moreover, the higher value found for *E*_d_ in comparison to *E*_a_ (35.33 kJ/mol) indicates that initiating denaturation of *Gs*XynA requires more energy than catalysis, a behavior previously reported for fungal xylanases as well [67].

The slight decrease in ΔH* with increasing temperature indicates that denaturation requires progressively less energy at higher temperatures [67]. A similar trend has been reported for other GH10 xylanases, including those from *Bacillus safensis* (ΔH* = 192.8–192.5 kJ·mol^−1^; 40–80 °C) [47], *Bacillus halodurans* TSEV1 (ΔH* = 251.7–251.5 kJ·mol^−1^; 65–80 °C) [62], and *Cohnella* sp. A01 (ΔH* = 230.07–229.86 kJ·mol^−1^) [68]. Overall, the high ΔH* values obtained for *Gs*XynA point to substantial structural rearrangements taking place upon thermal denaturation [65] and are consistent with its considerable thermostability. However, ΔH* values alone are not suitable indicators of enzyme stability. Protein stability arises from a delicate balance between stabilizing and destabilizing forces, governed by the interplay of disulfide bonds, hydrogen bonds, hydrophobic interactions, ionic contacts, and the overall degree of molecular folding. In this context, the Gibbs free energy of inactivation (ΔG*) is considered a more accurate and reliable parameter for assessing enzyme stability [47], as it integrates both enthalpic and entropic contributions. The progressive decline in ΔG* with increasing temperature reflects a gradual reduction in enzyme stability as thermal stress intensifies. These values demonstrate considerable enzyme stability and are comparable to those reported for endo-xylanases from *Thermotoga naphthophila* RKU-10^T^ (ΔG* = 116.92–115.73 kJ·mol^−1^; 96–98 °C) [60], *Bacillus safensis* (ΔG* = 103.1–99.9 kJ·mol^−1^; 60–80 °C) [47], *Bacillus halodurans* TSEV1 (ΔG* = 98.8 kJ·mol^−1^; 65 °C) [62], *Aspergillus niger* DFR-5 (ΔG* = 108.00–110.05 kJ·mol^−1^; 60–70 °C) [67], and *Cohnella* sp. A01 (ΔG* = 101.49–95.26 kJ·mol^−1^; 65–80 °C) [68].

The entropy of denaturation (ΔS*) which captures the degree of structural disorder gained as the enzyme transitions from its native ground state to the unfolding transition state, is another key thermodynamic parameter governing the extent of *Gs*XynA thermal inactivation. In fact, entropy generally augments with the denaturation of the protein [51]. The positive ΔS* values obtained for *Gs*XynA over the temperature range of 65–80 °C suggest that no considerable aggregation occurred during thermal inactivation, a trend previously reported for other bacterial endo-xylanases [47,62,68]. Taken together, the thermodynamic profile of *Gs*XynA thermal inactivation appears to be enthalpy-driven, with the high ΔH* values dominating over the entropic contribution across the entire temperature range studied. The observed decline in both ΔH* and ΔG* with increasing temperature, coupled with positive ΔS* values, is indicative of an enthalpy–entropy compensation phenomenon, in which the increasing molecular disorder upon unfolding only partially offsets the enthalpic stabilization, resulting in a gradual but progressive reduction in enzyme stability. This behavior has been previously described for other thermostable endo-xylanases [66] and further supports the considerable thermostability of *Gs*XynA under the conditions studied.

### 3.5. Structural Basis and Mechanism of GsXynA Thermal Denaturation

Given that *Gs*XynA belongs to the GH10 family, whose members share a canonical (α/β)_8_ TIM-barrel fold with a high α-helical content, CD spectroscopy was employed here both to confirm the expected secondary structure composition of the enzyme and to assess the thermal stability of its folded conformation. The secondary structure composition derived from the *Gs*XynA far-UV spectrum is in good agreement with that obtained from the crystal structure of IXT6 (PDB: 2Q8X), the closest structural homologue of *Gs*XynA (82% sequence identity), which showed 37.6% α-helix, 16.0% parallel β-sheet, 12.8% β-turns, and 33.6% random coil as determined by BeStSel analysis (Figure S5) [69]. Overall, the CD-derived secondary structure composition confirms that *Gs*XynA adopts a well-folded structure in solution, dominated by α-helical elements, as expected for a GH10 family endo-xylanase [8]. Having established this native conformation, the loss of the double-minimum CD signal upon heating can be interpreted as evidence of substantial disruption of the ordered secondary structure of *Gs*XynA, consistent with the protein precipitation observed during the thermal scan. This indicates that thermal denaturation is irreversible, as the protein neither recovers its native conformation upon cooling nor remains fully in solution. The apparent *T*_m_ obtained corroborates the optimal temperature and thermostability previously determined in biochemical assays. Interestingly, the *T*_m_ of *Gs*XynA was found to be higher than that reported for other GH10 xylanases, such as that of *Bacillus* sp. NG-27 [70] or that of *Bacillus* sp. TAR-1 [71].

The irreversible thermal denaturation of *Gs*XynA can be understood as a two-step process: a first step of reversible unfolding, followed by an irreversible alteration of the unfolded protein that leads to the final inactive state. Assuming this second step to be fast, the transition was described using an irreversible two-state model, in which the first-order kinetic constant varied with temperature according to the Arrhenius equation [56]. Supporting this model, the scan-rate dependence of the *T*_m_ observed for *Gs*XynA indicates that its thermal denaturation is kinetically controlled rather than a true equilibrium process. Within this kinetically controlled scenario, the higher activation energy of denaturation determined by CD (*E*_d_ = 567.08 kJ/mol), compared to that estimated from enzymatic inactivation (*E*_d_ = 307.085 kJ/mol), reflects the fact that these two values correspond to different processes. Under this kinetic model, the CD-derived *E*_d_ reflects the thermal unfolding process, typically coupled to protein aggregation, whereas the activity-derived *E*_d_ reflects the loss of catalytic function, assessed indirectly through residual activity measurements. These are therefore distinct, though related, observables of the same overall denaturation process, which are not necessarily governed by the same rate-limiting step. A comparable discrepancy between these two types of *E*_d_ values was previously reported by our group for a β-xylosidase [54]. The *E*_d_ value obtained from CD is, moreover, in agreement with results previously reported for xylanases from *Bacillus circulans* [72] and *Trichoderma reesei* [73]. Altogether, these findings indicate that the thermal unfolding of *Gs*XynA is an irreversible, kinetically controlled process, most likely driven by protein aggregation, a side process commonly underlying the irreversibility of protein thermal denaturation. Finally, further support for this interpretation comes from the marked increase in detector HT voltage recorded simultaneously with the CD signal during thermal denaturation, since changes in HT voltage are commonly used as an indirect indicator of sample turbidity arising from protein aggregation [74].

### 3.6. Inhibitory Effect and Mechanism of Phenolic Compounds on GsXynA Activity

The differential inhibitory potency observed among the six phenolic compounds tested can be interpreted in light of previous reports on xylanase inhibition. The moderate inhibition observed for acetosyringone, reaching approximately 20% at the highest concentration tested, is comparable to that reported for a xylanase from *A. oryzae* [75]. The stronger inhibition exerted by 4-hydroxybenzoic acid and *p*-coumaric acid is likewise consistent with previous findings: a similarly strong inhibition was reported for the GH10 endo-β-1,4-xylanase produced by *P. chrysogenum* [76]. Furthermore, 4-hydroxybenzoic acid has been reported to inhibit the xylanase from *Aspergillus terreus*, although to a lesser extent (~40% inhibition), while *p*-coumaric acid has also been described as an inhibitor of a xylanase from *Thermobacillus xylanilyticus* [14].

Given that 4HBAC emerged as the most potent inhibitor of *Gs*XynA, its mechanism of action was further examined. The parallel lines obtained in the Lineweaver-Burk plot, together with the reduction in both *K*_m_ and *V_max_* at increasing inhibitor concentrations and the near-constant *K*_m_/*V*_max_ ratio across concentrations, suggest that 4HBAC acts as an uncompetitive inhibitor on the enzyme, binding exclusively to the enzyme-substrate complex and forming a *Gs*XynA-xylan-4HBAC ternary complex. As a result, it is plausible to hypothesize that xylanase activity, as well as the release of products such as XOS, was affected. Few studies to date have characterized the mechanism underlying xylanase inhibition by phenolic compounds. Among them, Liu et al. [75] showed that acetosyringone inhibits the activity of a xylanase from *A. oryzae* in a reversible, noncompetitive manner, as determined through inhibition kinetics and conformational studies. Similarly, Hidayatullah et al. [77] reported noncompetitive inhibition of a commercial xylanase by vanillin. Non-competitive inhibition of xylanases by phenolic compounds has been associated with conformational changes in the enzyme structure. Boukari et al. [78] showed, through intrinsic fluorescence spectroscopy, that phenolic compounds inhibit a xylanase from *Thermobacillus xylanilyticus* through a non-competitive, multi-site mechanism, likely via surface residue interactions that induce steric inactivation. In line with this, Liu et al. [75] further reported that acetosyringone induces conformational changes in the *A. oryzae* xylanase, including local regional changes in the active-site pocket, without altering substrate affinity. While these studies describe non-competitive rather than uncompetitive inhibition, they support the notion that phenolic compounds can alter xylanase structure through surface interactions—a possibility that could be compatible with the uncompetitive behavior observed for 4HBAC, although this remains to be experimentally confirmed. To our knowledge, the present study is among the first to characterize the type of inhibition exerted by 4HBAC on a xylanase, identifying it as an uncompetitive inhibitor and providing a quantitative inhibition constant for this interaction.

Structural studies, such as fluorescence spectroscopy and molecular docking, could provide further insight into the interaction between *Gs*XynA and 4HBAC, findings that would be especially valuable given the comparatively limited attention paid to hemicellulase inhibition relative to cellulases. This would contribute to a better understanding of the factors negatively affecting lignocellulose deconstruction.

### 3.7. Hydrolysis Pattern and Synergistic Degradation of Beechwood Xylan by GsXynA and GsXynB2

The hydrolysis products generated by *Gs*XynA on beechwood xylan can be interpreted in the context of the enzyme’s mode of action. The presence of xylose suggested further hydrolysis of xylobiose and xylotriose or xylotetraose by *Gs*XynA, indicating that the enzyme acts in both an exo- and endo-acting manner. This hydrolysis pattern is consistent with that reported for other GH10 xylanases acting on beechwood or birchwood xylan. The recombinant XynB-A01 from *Cohnella* sp. A01, for instance, produced xylotetraose, xylobiose, and xylose as the main hydrolysis products, with no significant amounts of xylotriose, xylopentaose, or xylohexaose detected [68]. Similarly, the endoxylanase of *Gracilibacillus* sp. TSCPVG yielded XOS, xylobiose, and xylose as the principal final products of birchwood xylan hydrolysis after 24 h, as determined by HPLC [13]. The GH10 xylanase of *Geobacillus thermodenitrificans* TSAA1 displayed a comparable profile on birchwood xylan after short incubation times, as well as on wheat bran, corn cobs, and sugarcane bagasse after 12 h of incubation [48]. By contrast, the GH10 xylanase of *Geobacillus thermoleovorans* released xylobiose, xylotriose, xylotetraose, and xylopentaose from birchwood xylan shortly after the reaction began, whereas, on wheat bran, it produced xylose, xylobiose, and xylotriose within 2 h of incubation, with the concentrations of xylose and xylobiose continuing to rise up to 12 h of hydrolysis [49]. A similar behavior was observed for the GH10 xylanase XynDZ5, for which prolonged incubation with beechwood or oat-spelt xylans led to xylose as the main reaction product, followed by xylobiose, xylotriose and xylotetraose [79]. This progressive accumulation of xylose, together with the persistence of oligosaccharide bands, has generally been attributed to a secondary hydrolytic attack on xylooligosaccharide products by the same enzyme, supporting a mixed endo/exo-type mechanism rather than strict endo-specificity. Interestingly, the hydrolysis pattern exhibited by *Gs*XynA is also comparable to that of xylanases from other GH families. For example, the GH11 xylanase Xyn2A from *Trichoderma viride* released small amounts of xylose alongside the expected xylooligosaccharide products during xylan degradation [80]. Similarly, the GH30 xylanase *Ac*Xyn30B_12 from *Acetivibrio clariflavus* was shown to release a range of XOS, from xylopentaose to xylobiose, together with xylose, from partially acetylated birchwood xylan, confirming both its endo- and exo-acting mechanisms [81]. Altogether, these comparisons support the interpretation that *Gs*XynA operates primarily as an endo-xylanase but retains the capacity to further process XOS intermediates, releasing xylose as a minor but consistent product.

The complete degradation of xylan into monomeric sugars, acids, and monophenols requires the synergistic action of a consortium of xylanolytic enzymes [6,41]. Synergism between hemicellulolytic enzymes can be of two types: homeosynergy, which occurs between two enzymes that both act on the main chain or both act on side chains, and heterosynergy, which occurs between a side-chain and main-chain-cleaving enzymes [41]. Xylanases and β-xylosidases have been shown to act synergistically in xylan degradation [10,11,41], an effect that may enhance the enzymatic hydrolysis of xylan to xylose and contribute to cost reduction [82]. Having characterized the individual mode of action of *Gs*XynA, the synergistic potential of *Gs*XynA in combination with *Gs*XynB2 was next considered.

The very faint xylose spot and absence of detectable XOS produced by *Gs*XynB2 alone indicate that this β-xylosidase has limited capacity to act directly on polymeric xylan in the absence of an endo-xylanase to first generate shorter XOS substrates. The markedly more intense xylose spot and the visible reduction in the accumulation of XOS observed when both enzymes acted simultaneously clearly reflect the synergistic action of the two enzymes. The degree of synergy obtained in this work falls within the range of values reported for other binary xylanase/β-xylosidase systems. In this context, a synergy degree of approximately 1.7 was reported by Kakati et al. [10] for the combined action of the bifunctional exo-xylanase/β-xylosidase *Zg*GH43A with the endo-xylanase *Ac*Xyn30B_12. Similarly, Yang et al. [11] found relatively modest synergy degrees (0.98 and 1.16) when the GH43 bifunctional β-xylosidases/α-arabinosidases (Xyl43A and Xyl43B) acted simultaneously with the GH11 xylanase (Xyn11A) on beechwood xylan. Li et al. [82] reported synergy degrees comparable to those obtained in the present study for the simultaneous action of the GH11 endo-xylanase MxynB-8 and the GH39 β-xylosidase Xln-DT on poplar sawdust xylan, whereas Liu et al. [83] described even higher synergy degrees for the simultaneous hydrolysis of beechwood xylan by the GH43 β-xylosidase rXYL from *Bacillus pumilus* together with the GH11 endo-xylanase XynG1-1. Taken as a whole, these results support the cooperative relationship between *Gs*XynA and *Gs*XynB2 in beechwood xylan hydrolysis to produce xylose and XOS, highlighting their potential application in bioconversion processes such as biofuel production, as well as in the conversion of LCB into XOS.

## 4. Materials and Methods

All chemicals were of analytical grade and were used without further purification. Restriction enzymes and T4 DNA ligase were purchased from Roche Diagnostic S.L. (Biomol, Seville, Spain). Kapa-Hifi polymerase was from KapaBiosistems (Biomol, Seville, Spain). Primers were from Integrated DNA Technologies, IDT (Biomol, Seville, Spain). TALON TM metal affinity resin was purchased from Clontech Laboratories, Inc. (Biomol, Seville, Spain). Birchwood xylan, oat spelt xylan, and ortho-nitrophenyl xylopyranoside (*o*-NPX) were purchased from Sigma-Aldrich (St. Louis, MO, USA). 4-Nitrophenyl-β-D-xylopyranoside (*p*-NPX) and 3,5-dinitrosalicylic acid (DNSA) were obtained from Merck (Darmstadt, Germany) and Alfa Aesar (Ward Hill, MA, USA), respectively. Xylose was purchased from TCI (Tokyo, Japan). Beechwood xylan, wheat arabinoxylan, carboxymethylcellulose (CMC), 4-nitrophenyl-β-D-glucopyranoside (*p*-NPG), 4-nitrophenyl-β-xylobioside (*p*-NPX2), 4,6-*O*-(3-Ketobutylidene)-4-nitrophenyl-β-D-4^5^-glucosyl-xylopentaoside (*p*-NPXylX6), xylooligosaccharides (X–X6), and the D-xylose enzymatic assay kit were acquired from Megazyme (Bray, Ireland). 4-Hydroxybenzaldehyde was purchased from Fluka (Buchs, Switzerland), 4-hydroxyacetophenone and 4-hydroxybenzoic acid were purchased from Acros Organics (Geel, Belgium), vanillin was obtained from Alfa Aesar (Ward Hill, MA, USA), and acetosyringone and *p*-coumaric acid were purchased from Sigma-Aldrich (St. Louis, MO, USA). β-xylosidase from *G. stearothermophilus* CECT43 (*Gs*XynB2), used in the synergistic studies, was obtained as previously described [54].

### 4.1. Microorganism and Culture Conditions

*G. stearothermophilus* CECT43, acquired from the Spanish Type Culture Collection (CECT), was used as a possible donor of the endo-1,4-beta-xylanase gene (*xyna*). The strain was grown at 55 °C for 20 h in a Luria–Bertani medium (LB) (1% tryptone, 0.5% yeast extract, 0.5% NaCl, pH 7.2). *E. coli* DH5*α* was used to clone the gene *xyna*, and *E. coli* BL21 (DE3) was used as a host to overexpress the recombinant protein.

### 4.2. Cloning of Endo-1,4-Beta-Xylanasegene (xyna) Gene

A single-colony isolate of *G. stearothermophilus* CECT43 was chosen for DNA isolation using a boiling procedure [84]. A supernatant aliquot of 5 µL containing genomic DNA of the strain was used to amplify the gene encoding for putative *xyna* by PCR. The primers used to amplify the gene from *G. stearothermophilus* CECT43 (*gsxyna*) were designed based on GenBank sequence accession number D28121.1 [34]. These were GstXynABam5 (5′-GGATCCGTGACAGGAAAGAGGAATGTGCTCATCCATCC-3′) and GstXynAXho3 (5′-CTCGAGGATATTCACCACCCGCCAAAAAGC-3′) including *Bam*HI and *Xho*I restriction sites, respectively.

The PCR product was purified from an agarose gel using the GeneJET Gel Extraction Kit (Thermo Fisher Scientific, Waltham, MA, USA), treated with *Bam*HI and *Xho*I enzymes (New England Biolabs, Ipswich, MA, USA). The digested fragment was ligated into pET-21b(+) plasmid (Novagen, Millipore Sigma, Merck KGaA, Darmstadt, Germany) cleaved with the same enzymes to create plasmid pJMC118. The sequence of the gene was confirmed by sequencing using an Applied Biosystems 3500 Series Genetic Analyzer (Thermo Fisher Scientific, Waltham, MA, USA) at the Nucleic Acids Analysis Service of the University of Almería as previously described [51].

### 4.3. Expression and Purification of the GsXynA

The protocol was similar to that described in [84] with minor changes. The *E. coli* BL21 (DE3) strain, transformed with the plasmid pJMC118, was grown in an LB medium supplemented with ampicillin. For induction and expression of the gene, isopropyl-*β*-*D*-1-thiogalactopyranosidase (IPTG) was added to 800 mL of the LB medium (0.1 mM final concentration) and incubated at 32 °C overnight. The cells were collected by centrifugation (Beckman J-26 XPI centrifuge) (6500× *g*, 4 °C, 25 min) (Beckman Coulter, Brea, CA, USA) and stored at −20 °C until further processing.

The cell walls were disrupted on ice by sonication as described before [52]. The recombinant enzyme was purified by immobilized metal affinity chromatography (IMAC) using cobalt resin as previously described [85], with the addition of 1 mM 2-mercaptoethanol. Imidazole was removed by ultrafiltration using a Vivaspin 20 mL ultrafiltration tube (Sartorius, Gotinga, Germany) with a 10,000 Da molecular weight cut-off membrane. Gel filtration chromatography with a Superdex 200 gel filtration column (Cytiva, Marlborough, MA, USA) was carried out to eliminate any DNA or co-eluted proteins [86]. The purified enzyme was concentrated using Vivaspin concentrator (Sartorius, Gotinga, Germany), dialyzed against 100 mM sodium phosphate buffer, pH 6.5, and stored at 4 °C until use. Protein concentration was determined from the absorbance at 280 nm using the predicted extinction coefficient of the protein, 68,535 M^−1^ cm^−1^ [87].

### 4.4. Purity and Molecular Mass Analysis in Denaturing and Native Conditions

The estimated molecular mass and purity of the protein at different stages of the purification were assessed by SDS-PAGE using 15% polyacrylamide gels, followed by Coomassie Brilliant Blue R-250 staining and destaining. Size exclusion chromatography–FPLC (SEC-FPLC) analysis was performed to determine the molecular mass of the native protein. *Gs*XynA (150 µL, 0.75 mg/mL in 20 mM Tris-HCl pH 8.0) was loaded onto a Tricorn Superdex 75 gel-filtration column (GE Healthcare, Chicago, IL, USA) in an AKTA-purifier FPLC system (GE Healthcare, Chicago, IL, USA) using 20 mM Tris-HCl pH 8.0 with 150 mM NaCl as the running buffer. Ovoalbumin (~40 and ~80 kDa) and carbonic anhydrase (~29 kDa) were used as standards.

### 4.5. Determination of Enzymatic Activity and Substrate Specificity of GsXynA

The enzymatic activity of *Gs*XynA was evaluated against a range of commercial xylan substrates, including beechwood xylan, birchwood xylan, arabinoxylan, oat xylan, and corn xylan, as well as carboxymethylcellulose as a cellulose substrate. The standard reaction consisted of pre-incubating 25 µL of 1 µM enzyme with 50 µL of 0.1 M sodium phosphate buffer (pH 6.5) for 1 min at 50 °C, followed by addition of 25 µL of the corresponding substrate (1%, *w*/*v* final concentration). The reaction mixture was incubated at 50 °C and 250 rpm for 5 min, after which the released reducing sugars were quantified by the DNS colorimetric method [88], using a D-xylose standard curve. One unit of xylanase activity was defined as the amount of enzyme required to release 1 μmol of xylose per min. *Gs*XynA activity was also assayed using *p*-NPXylX6, *p*-NPX2, *p*-NPX, *o*-NPX, and *p*-NPG, all at a final concentration of 1 mM. Reactions were incubated for 5–10 min at 50–70 °C and stopped with 100 µL of 1 M Na_2_CO_3_ (or 2% Tris buffer (pH 10) for *p*-NPXylX6). The concentration of released *p*-nitrophenol was determined by measuring the absorbance at 410 nm (ε = 18.1 mM^−1^·cm^−1^).

### 4.6. Biochemical Characterization of GsXynA

All characterization assays were performed using beechwood xylan as the substrate and were performed in triplicate.

Effect of pH on activity and pH stability of *Gs*XynA. The optimum pH of *Gs*XynA was determined by measuring enzymatic activity across a pH range of 1.92 to 11.9, using the following 100 mM buffer systems: formate (pH 1.92–2.93), formic acid (pH 3.0–4.0), acetate (pH 4.0–5.51), phosphate (pH 5.8–7.0), Tris (pH 7.0–9.25), carbonate (pH 9.42–10.93), and phosphate (pH 11.3–11.9). Overlapping buffers were used to verify that the activity was due solely to pH and not to the type of ion present. Beechwood xylan was prepared at 2% (*w*/*v*) in each of the corresponding buffers. Reactions were performed by incubating 25 µL of 1 µM enzyme with 25 µL of the appropriate buffer and 50 µL of 2% xylan dissolved in the above-mentioned buffers for 5 min at 55 °C. Reducing sugars released were subsequently quantified by the DNS colorimetric method, as described in Section 4.5. For pH stability assessment, *Gs*XynA at 1 µM was incubated for 12 h at 4 °C in each of the buffers spanning pH 1.92–11.9. Following incubation, 25 µL of the enzyme-buffer mixture were combined with 50 µL of 0.1 M phosphate buffer (pH 6.5) and incubated for 1 h at 4 °C to allow activity recovery. The sample was then pre-incubated for 1 min at 55 °C, and residual activity was measured under standard assay conditions.

Effect of temperature on activity and thermostability of *Gs*XynA. The optimum temperature of *Gs*XynA was determined by measuring enzymatic activity across a temperature range of 4 to 90 °C. At each temperature, 25 µL of 1 µM enzyme were pre-incubated with 50 µL of 0.1 M sodium phosphate buffer (pH 6.5) for 1 min, after which 25 µL of 4% beechwood xylan were added and the reaction was allowed to proceed for 5 min. Reactions were stopped on ice and the enzyme activity was measured by estimating the released reducing sugars (Section 4.5). For thermostability assessment, 75 µL of 0.33 µM *Gs*XynA in 0.1 M sodium phosphate buffer (pH 6.5) were pre-incubated for 1 h at temperatures ranging from 4 to 90 °C. Following pre-incubation, samples were equilibrated for 2 min at room temperature and subsequently pre-warmed for 1 min at 70 °C. Substrate was added and the reaction was allowed to proceed for 5 min. Residual activity was quantified as mentioned earlier in Section 4.5. These residual activity data were then fitted to a sigmoidal (Boltzmann) equation using SigmaPlot^®^ (version 12.0, Systat Software, Inc., San Jose, CA, USA). The half-inactivation temperature was defined as the temperature at which enzyme activity was reduced to 50% of the value obtained for the untreated control.

### 4.7. Determination of K_m_, V_max_, and K_cat_ of GsXynA

Kinetic parameters of *Gs*XynA were determined using beechwood xylan, birchwood xylan, arabinoxylan, *p*-NPXylX6, and *p*-NPX2. Assays were performed in triplicate in 0.1 M phosphate buffer (pH 6.5) at a fixed enzyme concentration (25 nM) over a range of substrate concentrations, namely 0–3% for xylan substrates, 0–1.5 mM (0–1.6 mg/mL) for *p*-NPXylX6, and 0–4 mM (0–1.6 mg/mL) for *p*-NPX2, following the reactions conditions described above for each substrate type. The date obtained were fitted to the Michaelis–Menten equation by non-linear regression analysis using SigmaPlot^®^ (version 12.0, Systat Software, Inc., San Jose, CA, USA). The turnover number (*k*_cat_) was calculated by dividing *V*_max_ by the enzyme concentration, and catalytic efficiency was expressed as the ratio of *k*_cat_ to *K*_m_ (*k*_cat_/*K*_m_).

To investigate the thermodynamic of beechwood xylan hydrolysis by *Gs*XynA, *V*_max_ was determined at temperatures ranging from 50 to 75 °C, with assays likewise performed in triplicate. The activation energy (*E*_a_) of the catalytic reaction was subsequently calculated from the slope of an Arrhenius plot of ln *V*_max_ against 1/T [65].

### 4.8. Thermal Inactivation Kinetics

The thermal inactivation of *Gs*XynA was determined by incubating the enzyme in 0.1 M phosphate buffer (pH 6.5) at 65, 70, 75, and 80 °C in the absence of substrate. Pre-incubation times vary from 5 min to 30 min at the highest temperatures and from 1 to 18 h at the lowest ones. Aliquots were drawn at the desired intervals and immediately cooled for 1 min at 4 °C to halt further thermal inactivation, after which residual enzymatic activity was subsequently measured under standard assay conditions. The inactivation rate constant (*K*_d_) was determined from the slope of the pseudo-first-order plot of ln(A/A_0_) versus time at each temperature tested [65]. The half-life of *Gs*XynA at each temperature was calculated using the following equation:

(1) $$t_{1/2}=\frac{1}{K_{d}}\ln\left(2\right),$$

The value of *D*, defined as the time required to achieve a 90% reduction in initial activity, was obtained according to the following expression:

(2) $$D=\frac{1}{K_{d}}\ln\left(10\right),$$

The activation energy of denaturation of *Gs*XynA (*E*_d_) was obtained from the slope of the Arrhenius plot of ln *K*_d_ against the reciprocal of the absolute temperature (1/T). Finally, the changes in free energy (ΔG*), enthalpy (ΔH*), and entropy (ΔS*) during thermal denaturation of the enzyme were obtained from the equations:

(3) $$∆G^{*}=\;-RT\ln\left(\frac{K_{d}h}{K_{B}T}\right),$$

(4) $$∆H^{*}=E_{d}-RT,$$

(5) $$∆S^{*}=\frac{{∆H}^{*\;}-∆G^{*}}{T},$$

where *h* is the Planck constant (6.626·10^−34^ J·s) and *k_B_* is the Boltzmann constant (1.381·10^−23^ J·K^−1^).

### 4.9. Circular Dichroism Analysis

Circular dichroism (CD) experiments were performed on a Jasco J815 spectropolarimeter (Tokyo, Japan) equipped with a thermostated cell holder and interfaced with a Peltier accessory. Far-UV spectra of *Gs*XynA, in its native or denatured form, were recorded at 4 μM protein in a 0.1-cm-pathlength cell, over a wavelength range of 260 to 190 nm. Spectra were acquired at a scan rate of 50 nm/min, with a response time of 2 s, and averaged over five scans at 25 °C. The instrument was continuously flushed with nitrogen at a flow rate of 10 L/min. The secondary structure of *Gs*XynA was predicted using the CDNN deconvolution software [55].

The thermal transition of 4 µM *Gs*XynA in phosphate buffer at pH 6.5 was recorded at 222 nm using a 0.1-cm-pathlength cell over a temperature range of 55 to 95 °C, at scan rates of 0.4, 0.6, 0.8, 1.0, and 1.5 °C/min, and with a response time of 8 s. Precipitation was observed at all five heating rates. Thermal unfolding data were analyzed using a two-state model [52]. The observed raw ellipticity (*S*_obs_) was described as a function of temperature according to the following equation:

(6) $$S_{obs}=\frac{S_{N}+S_{U\;}exp(-\frac{{∆H}_{VH}}{R}\left(\frac{1}{T}-\frac{1}{T_{m}}\right))}{1+exp(-\frac{{∆H}_{VH}}{R}\left(\frac{1}{T}-\frac{1}{T_{m}}\right))},$$

where *S*_N_ = *A*_N_ + *B*_N_*T* and *S*_U_ = *A*_U_ + *B*_U_*T* represent the linear baselines of the native (N) and unfolded (U) states, with slopes *B*_N_ and *B*_U_ and intercepts *A*_N_ and *A*_U_, respectively. Δ*H*_VH_ is the apparent van’t Hoff enthalpy change, *R* is the universal gas constant, *T* is the absolute temperature (K), and *T_m_* is the melting temperature, defined as the midpoint of the thermal transition at which the protein population is equally distributed between the native and unfolded states. All curve fitting was performed using KaleidaGraph (version 3.5, Synergy Software).

The dependence of *T*_m_ on the scan rate was analyzed using the Arrhenius-based approach described by Sanchez-Ruiz et al. [56], in which ln(*V*/*T*_m_^2^) is plotted against 1/*T*_m_. The activation energy of denaturation (*E*_d_) was obtained by fitting the data to the following equation:

(7) VTm2=AREde−EdRTm,

where *V* is the scan rate (K/min), *T*_m_ is the transition midpoint temperature (K), *A* is the pre-exponential frequency factor, and *R* is the universal gas constant.

### 4.10. Effect of Phenolic Compounds on GsXynA Activity

The effect of phenolic compounds on *Gs*XynA activity was determined by measuring enzymatic activity in the presence of 4-hydroxybenzaldehyde, vanillin, 4-hydroxyacetophenone, acetosyringone, 4-hydroxybenzoic acid, and *p*-coumaric acid, each tested at three concentrations (1.5, 2.5, and 5 mg/mL). All compounds were dissolved in DMSO and added to the reaction mixture during the enzyme pre-incubation step, with a final DMSO concentration not exceeding 5% (*v*/*v*) in any assay. A control group containing 5% (*v*/*v*) DMSO alone, without any phenolic compound, was included under the same reaction conditions to evaluate the effect of the solvent itself on enzyme activity. Reactions were performed at 75 °C for 3 min at final concentrations of 150 nM *Gs*XynA and 1% (*w*/*v*) beechwood xylan as substrate, and reducing sugars were quantified by the DNS colorimetric method as described above.

The mode of inhibition of 4-hydroxybenzoic acid (4HBAC) was characterized using classical Michaelis–Menten kinetics. The enzymatic activity of *Gs*XynA was measured in the absence and presence of 4HBAC (at concentrations ranging from 0 to 4 mg/mL), using beechwood xylan at varying concentrations. The apparent kinetic parameters (*V*_maxi_ and *K*_mi_) were determined from Lineweaver–Burk double-reciprocal plots. The uncompetitive inhibition constant (*K_ui_*) was obtained by secondary plot analysis, in which the y-intercepts of the Lineweaver–Burk plots were replotted against the corresponding inhibitor concentrations and the *K*_ui_ value was determined from the x-intercept by linear regression [53].

To assess whether the inhibition exerted by 4HBAC was reversible, *Gs*XynA was pre-incubated with 5 mg/mL 4HBAC overnight at 4 °C. The mixture was then dialyzed in 0.1 M phosphate buffer (pH 6.5) using Vivaspin 500 centrifugal filtration units (Merck Millipore) to remove the unbound inhibitor. Residual enzymatic activity was subsequently measured to evaluate the extent of activity restoration and to characterize the binding nature of 4HBAC to the enzyme.

### 4.11. Thin-Layer Chromatography (TLC) Analysis of the Hydrolytic Products

To determine the hydrolysis products from beechwood xylan by *Gs*XynA, a reaction mixture (100 µL) containing 250 nM enzyme and 1% (*w*/*v*) beechwood xylan in 100 mM phosphate buffer (pH 6.5) was incubated at 70 °C for 12 h. After enzymatic hydrolysis, the samples were inactivated by immersion in an ice bath. One microlitre of sample was spotted on Silica Gel 60 F254 TLC plates (Merck, Darmstadt, Germany), and the plates were developed in a chloroform:acetic acid:distilled water (3:6:1 *v*/*v*/*v*) solvent system. The plates were air-dried at room temperature, soaked in staining solution (methanol:sulfuric acid, 9:1 *v*/*v*, containing 0.2% orcinol), and heated at 85 °C until the spots were clearly visible [61]. Xylose (X1), xylobiose (X2), xylotriose (X3), xylotetraose (X4), xylopentaose (X5), and xylohexaose (X6) served as reference standards.

### 4.12. Synergy Between GsXynA and GsXynB2

The synergistic interaction between the xylanase *Gs*XynA and the β-xylosidase *Gs*XynB2—previously cloned and characterized from *Geobacillus stearothermophilus* CECT43 by our group [54]—was evaluated using beechwood xylan as a substrate. The enzyme protein mass ratios studied were 100:0, 33:67%, and 0:100 xylanase to β-xylosidase. All reactions were carried out at 70 °C in 0.1 M phosphate buffer (pH 6.5). After 0, 4 and 24 h of incubation, the mixtures were kept in an ice bath to stop the reaction. The released D-xylose was determined using an enzymatic kit (Megazyme, Bray, Ireland). The hydrolysis products were also analyzed using TLC as described in Section 4.11.

The degree of synergy (DS) was calculated using the following equation:

(8) $$DS=\;\frac{Y_{1+2}}{Y_{1}\;+\;Y_{2}},$$

where Y_1+2_ is the xylose released when *Gs*XynA and *Gs*XynB2 were both present, Y_1_ is the xylose released by *Gs*XynA alone, and Y_2_ that released by *Gs*XynB2 alone.

Saccharification yield of xylan during sinergism was determined by using the following equation:

(9) $$Percentage\;of\;xylan\;hydrolysis\;=\;\frac{D-xylose\;produced}{D-xylose\;content\;in\;xylan\;}\;\times \;100,$$

Theoretical D-xylose content in xylan was estimated considering that the D-xylose content of beechwood xylan is 79.9% *w*/*w* [89].

## 5. Conclusions

Here we reported the identification, heterologous expression, and comparative biochemical, thermodynamic, and structural characterization of *Gs*XynA, a novel GH10 endo-β-1,4-xylanase from the thermophilic bacterium *Geobacillus stearothermophilus* CECT43, together with an assessment of its inhibition by phenolic compounds and its synergistic action with the β-xylosidase *Gs*XynB2.

*Gs*XynA shares strict active-site conservation and a monomeric behavior with other characterized intracellular GH10 xylanases such as IXT6, reflecting strong evolutionary pressure to preserve both structural integrity and catalytic function within this enzyme subfamily. Its broad substrate range, alkaline tolerance, and wide thermal activity profile, together with its low cellulase activity, place *Gs*XynA among the most versatile bacterial GH10 xylanases described to date, supporting its suitability for applications requiring strict xylan specificity. The hydrolysis pattern observed, dominated by xylobiose and xylotetraose alongside minor xylose release, further indicated that *Gs*XynA acts through a mixed endo/exo mechanism rather than strict endo specificity, a behavior consistent with that reported for related GH10 and other glycoside hydrolase family xylanases. Regarding thermal behavior, the gradual, temperature-dependent loss of activity and the high inactivation energy indicated that *Gs*XynA undergoes an irreversible, kinetically controlled unfolding process, most likely driven by aggregation, in which catalytic function is lost before the secondary structure is substantially disrupted. The thermostability compares favorably with, and in several cases exceeds, that reported for other GH10 xylanases, suggesting that *Gs*XynA could represent a promising scaffold for future protein engineering efforts aimed at further enhancing its stability, a potential that remains to be experimentally validated through future mutagenesis studies. The characterization of 4-hydroxybenzoic acid as an uncompetitive inhibitor of *Gs*XynA presented in this work contributes new insight into an underexplored aspect of hemicellulase regulation, with direct relevance to the factors that may negatively affect lignocellulose deconstruction. Finally, the clear synergistic cooperation between *Gs*XynA and *Gs*XynB2, yielding degrees of synergy consistent with other xylanase/β-xylosidase systems, confirmed the complementary roles of these enzymes in the complete conversion of xylan into D-xylose and XOS.

Taken together, these findings support *Gs*XynA as a thermostable, versatile, and mechanistically well-characterized endo-xylanase with potential for industrial biocatalysis, particularly within enzymatic cocktails for the saccharification of LCB and the production of XOS, with prospective applications extending to biofuel production and other xylan-based bioconversion processes relevant to a circular bioeconomy.

## Figures and Tables

**Table 1:** Specific activity of *Gs*XynA on different substrates.

Substrate	Specific Activity (U·mg^−1^)
Beechwood xylan (1%)	248.27 ± 6.96
Birchwood xylan (1%)	309.22 ± 40.10
Oat xylan (1%)	236.56 ± 18.38
Wheat arabinoxylan (1%)	398.64 ± 67.50
CMC (1%)	17.70 ± 1.14
*p*-NPXylX6 (1 mM)	11.61 ± 1.70
*p*-NPX2 (1 mM)	43.46 ± 2.20
*p*-NPX (1 mM)	1.09 ± 0.08
*o*-NPX (1 mM)	6.39 ± 3.97
*p*-NPG (1 mM)	0.09

**Table 2:** Kinetic parameters of *Gs*XynA * with different substrates.

Substrate	*K*_m_ (mg/mL)	*V*_max_ (µmol/min)	*k*_cat_ (s^−1^)	*k*_cat_/*K*_m_ (s^−1^·mg^−1^·mL)
Beechwood xylan	3.24 ± 0.82	0.108 ± 0.010	718.66	221.80
Birchwood xylan	3.36 ± 0.62	0.042 ± 0.003	280.00	83.33
Wheat arabinoxylan	3.41 ± 0.56	0.059 ± 0.003	395.33	115.93
*p*-NPXylX6	0.56 ± 0.13 mg/mL 0.53 ± 0.12 mM	0.004 ± 0.0004	24.67	44.05 s^−1^·mg^−1^·mL 46.55 s^−1^·mM^−1^
*p*-NPX2	0.06 ± 0.01 mg/mL 0.15 ± 0.02 mM	0.005 ± 0.0003	32.67	544.50 s^−1^·mg^−1^·mL 217.80 s^−1^·mM^−1^

* *Gs*XynA concentration in the assays was 25 nM.

**Table 3:** Kinetic and thermodynamic parameters for the thermal inactivation of *Gs*XynA. *E*_d_ = 307.08 kJ·mol^−1^.

*T* (K)	*K_d_* (min^−1^)	*R* ^2^	*t*_1/2_ (min)	*D* (min)	ΔH* (kJ·mol^−1^)	ΔG* (kJ·mol^−1^)	ΔS* (J·mol^−1^·K^−1^)
338	0.0011	0.986	630.1	2093.3	304.28	113.78	563.59
343	0.0060	0.982	115.0	383.8	304.23	110.66	564.37
348	0.0336	0.974	20.6	68.5	304.19	107.34	565.67
353	0.1075	0.990	6.4	21.4	304.15	105.51	562.72

**Table 4:** Kinetic parameters of *Gs*XynA in the absence and presence of 4HBAC.

	*K*_m_ (mg/mL)	*V*_max_ (mmol/min)	*K*_m_/*V*_max_ (mg·min·mmol^−1^·mL^−1^)	*K*_iu_ (mg/mL)
*Gs*XynA *	3.51	4.76·10^−4^	7384.64	2.75
*Gs*XynA * + 4HBAC (2.5 mg/mL)	2.59	3.32·10^−4^	7810.14	
*Gs*XynA * + 4HBAC (3 mg/mL)	2.17	2.62·10^−4^	8276.01	
*Gs*XynA * + 4HBAC (4 mg/mL)	1.89	2.01·10^−4^	9402.66	

* *Gs*XynA concentration in the assays was 150 nM.

**Table 5:** Enzymatic hydrolysis of beechwood xylan by *Gs*XynA and *Gs*XynB2 alone or in combination.

Enzyme System	Reaction Time (h)	[D-Xylose] ^a^ (mg/mL)	Degree of Synergy	Yield (%)
*Gs*XynA	4	0.58	-	7.26
	24	0.84	-	10.63
*Gs*XynB2	4	0.15	-	1.86
	24	0.28	-	3.45
*Gs*XynA + *Gs*XynB2	4	3.12	4.28	39.06
	24	4.25	3.79	53.23

^a^ The concentrations of xylose released were determined using the D-Xylose Assay Kit (Megazyme, Bray, Ireland).

## Data Availability

The data presented in this article are available from the authors upon requirement.

## Supplementary Materials

The following supporting information can be downloaded at: https://www.mdpi.com/article/10.3390/ijms27167339/s1.

## Abbreviations

The following abbreviations are used in this manuscript:

LCB	Lignocellulosic biomass
GH	Glycoside hydrolase
XOS	Xylooligosaccharides
CECT	Spanish Type Culture Collection
SEC-FPLC	Size exclusion chromatography-FPLC
CMC	Carboxymethylcellulose
*E*_a_	Activation energy
*K*_d_	Inactivation rate constant
*E*_d_	Activation energy of denaturation
4HBAC	4-hydroxybenzoic acid
CD	Circular dichroism
LB	Luria–Bertani medium
IPTG	Isopropyl-*β*-*D*-1-thiogalactopyranosidase
IMAC	Immobilized metal affinity chromatography
TLC	Thin-layer chromatography
