# Supplementary material for: Endo-β-1,4-Xylanase GH10 from Geobacillus stearothermophilus CECT43: Biochemical Characterization, Insights into Its Thermal Behavior, and Synergistic Effects with a β-Xylosidase
Source: Int J Mol Sci. 2026 Aug 17;27(16):7339. doi: 10.3390/ijms27167339 (PMC13513542; doi:10.3390/ijms27167339)
Supplement: Supplementary file 1 [file ijms-27-07339-s001.zip › ijms-4491605-supplementary.pdf]

Article

Endo-β-1,4-Xylanase GH10 from *Geobacillus stearothermophilus* CECT43: Biochemical Characterization, Insights into Its Thermal Behavior, and Synergistic Effects with a β-Xylosidase

Alba Carrillo-Moreno [1,2,†], María Gabriela Álvarez-Rodríguez [3,†], Sergio Martínez-Rodríguez [4,5], Pablo Soriano-Maldonado [1,2], Josefa María Clemente-Jiménez [1,2], Francisco Javier Las Heras-Vázquez [1,2,*] and Lellys M. Contreras [1,2,*]

[1] Departamento Química y Física, Universidad de Almería, 04120 Almería, Spain; albacarrillo@ual.es (A.C.-M.); pablo.soriano@ual.es (P.S.-M.); jmclemen@ual.es (J.M.C.-J.)
[2] CIAMBITAL, CeiA3, University of Almería, 04120 Almería, Spain
[3] Instituto de Biotecnología Sanitaria de Elche (IDIBE), Universidad Miguel Hernández, 03202 Elche, Spain; m.alvarezr@umh.es
[4] Departamento Bioquímica y Biología Molecular III e Inmunología, Universidad de Granada, 18071 Granada, Spain
[5] Raw Materials, Human and Environmental Health, UGR, Associated Unit of the CSIC by the IACT-CSIC, 18100 Granada, Spain
* Correspondence: fjheras@ual.es (F.J.L.H.-V.); mariela@ual.es (L.M.C.); Tel.: +34-950-015055 (F.J.L.H.-V.); +34-950-015615 (L.M.C.)
† These authors contributed equally to this work.

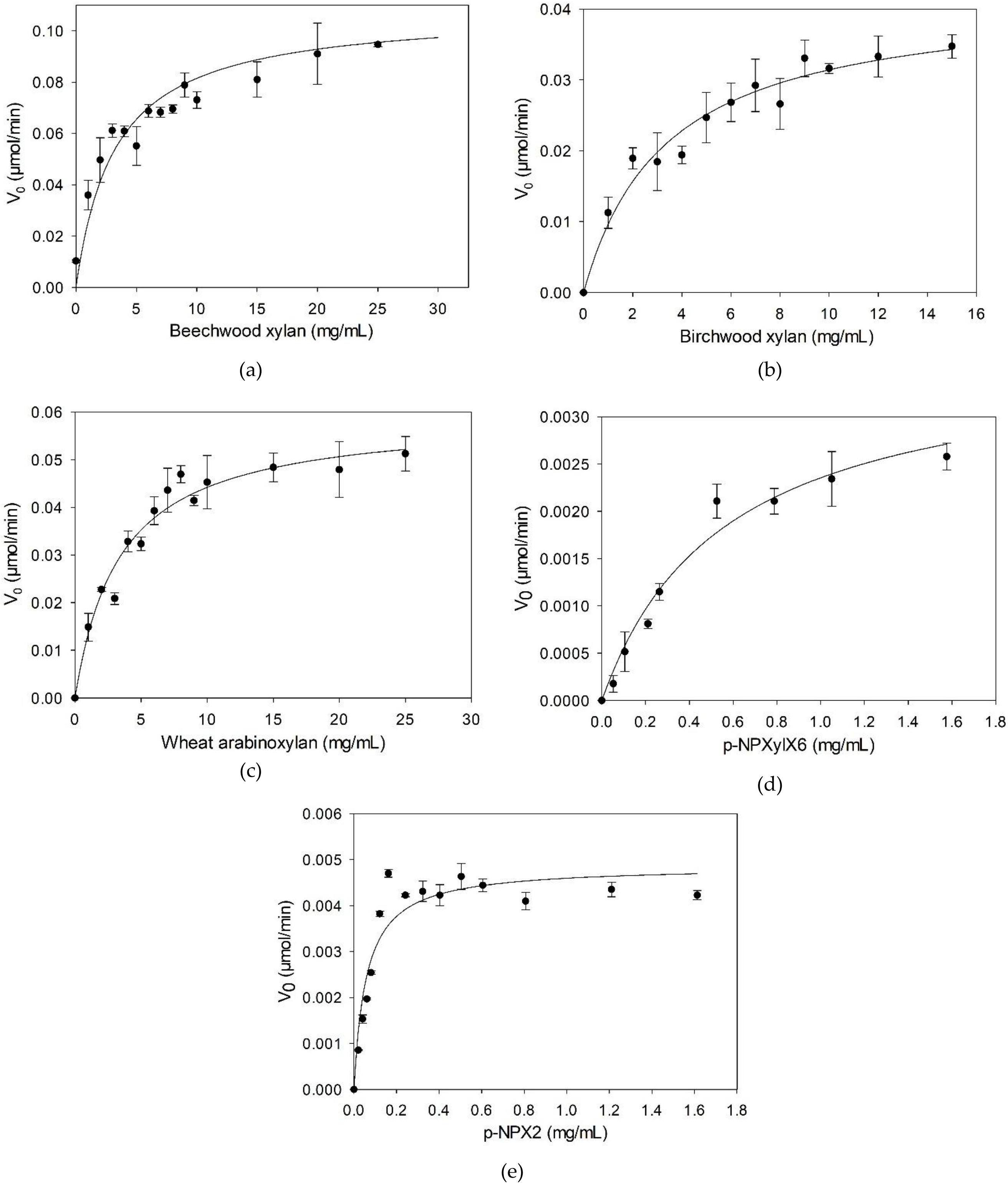

Figure S1. Michaelis–Menten plots for *Gs*XynA with (a) beechwood xylan, (b) birchwood xylan, (c) wheat arabinoxylan, (d) *p*-NPXylX6, and (e) *p*-NPX2 as substrates.

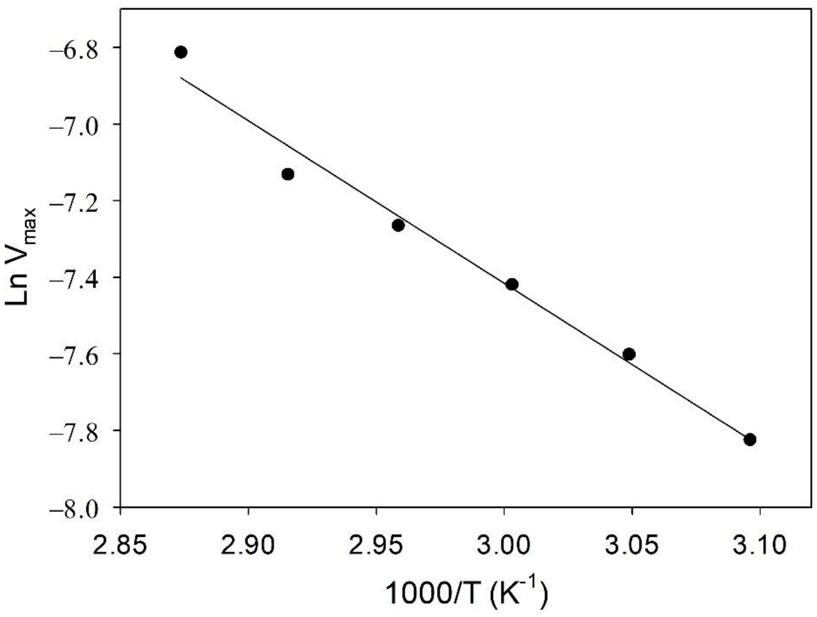

Figure S2. Arrhenius plot for the determination of the activation energy (E_a) of beechwood xylan hydrolysis. V_{max} values was determined at different temperatures (50–75 °C), and E_a was calculated from the slope of a plot of ln V_{max} vs. 1000/T. The fit yielded R^2 value of 0.98.

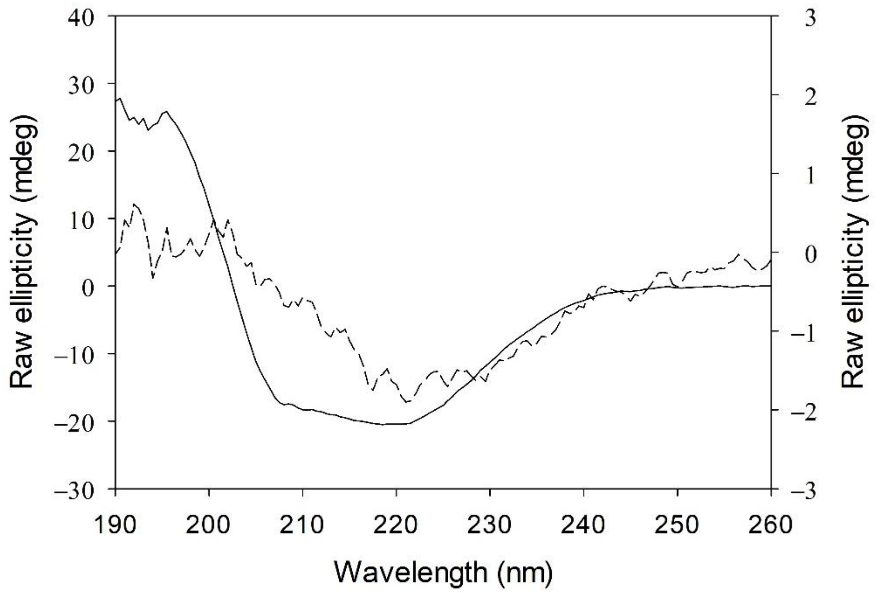

(a)

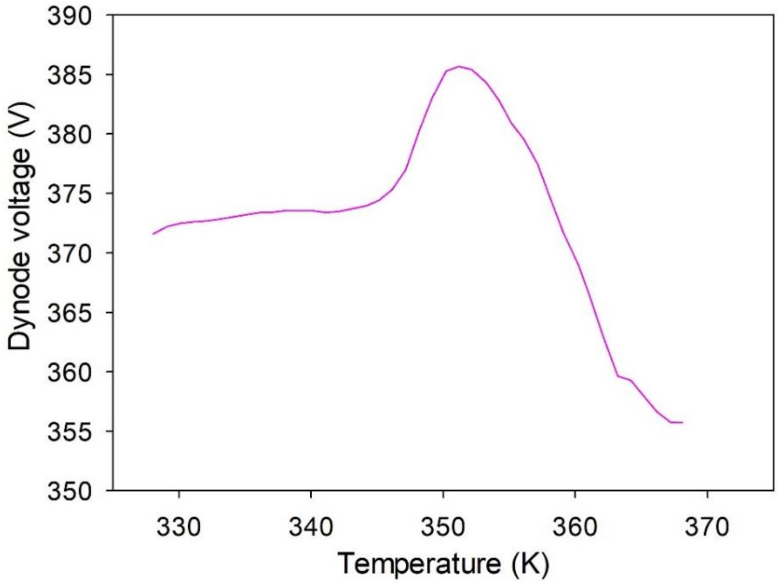

(b)

Figure S3. (a) Far-UV CD spectra of *Gs*XynA in its native and thermally denatured states**.** The CD spectrum recorded at 25 °C prior to thermal treatment (solid line, left y-axis) represents the native conformation of the enzyme. The CD spectrum recorded at 25 °C after a thermal scan from 55 to 95 °C followed by cooling to 4 °C (dashed line, right y-axis) represents the thermally denatured state of *Gs*XynA. Visible protein precipitation was observed upon completion of the thermal scan, indicating that thermal denaturation of *Gs*XynA is irreversible under these conditions. The use of dual y-axes reflects the difference in signal intensity between the native and denatured forms, consistent with the substantial loss of ordered secondary structure and protein content in solution following thermal treatment. Spectra were recorded over the wavelength range of 190–260 nm using a 0.1 mm cuvette. (b) Dynode voltage melting curve, recorded at 222 nm simultaneously with the CD data shown as the magenta curve in Figure 5A. The reduction in voltage observed for *Gs*XynA upon heating above 78 °C reflects precipitation of the heat-unfolded protein.

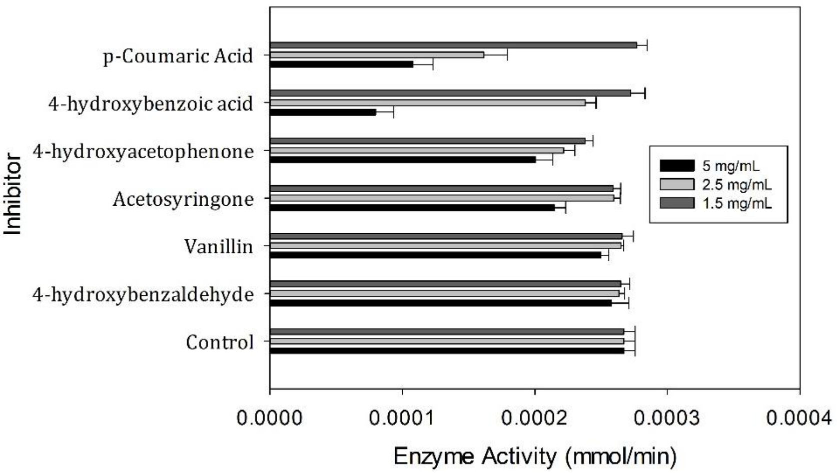

(a)

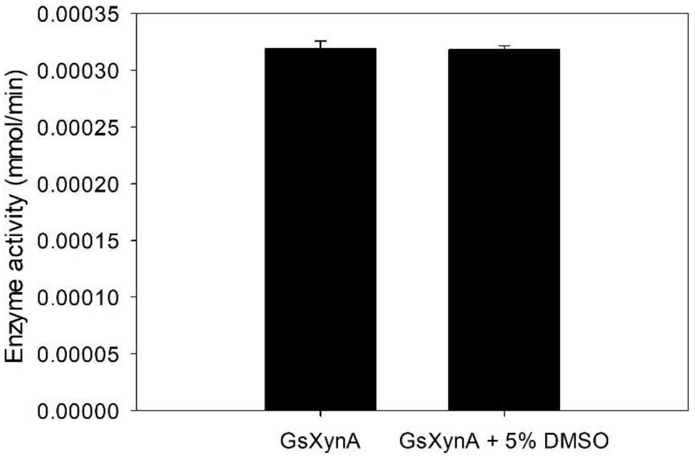

(b)

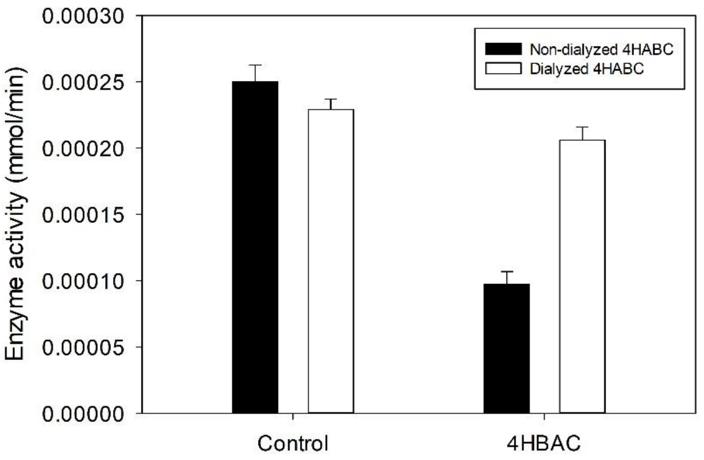

(c)

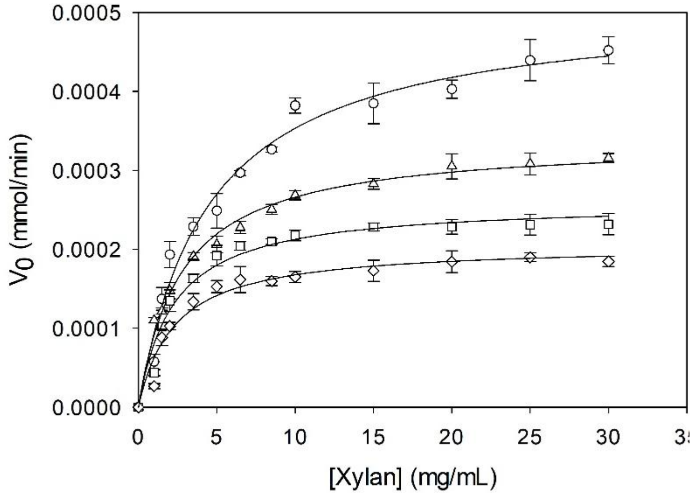

(d)

Figure S4. Effect of phenolic compounds and DMSO on *Gs*XynA activity, and characterization of inhibition reversibility and kinetics. (a) *Gs*XynA activity in the presence of different concentrations of phenolic compounds. (b) Effect of DMSO on *Gs*XynA activity. The enzyme was pre-incubated with 5% (*v*/*v*) DMSO at 4 °C for 1 h prior to the enzymatic reaction, performed under standard conditions. (c) Reversibility of the inhibition by 4HBAC at 5 mg/mL. (d) Michaelis-Menten curve of *Gs*XynA in the absence (circles) and presence of 4HBAC: 2.5 mg/mL (triangles), 3 mg/mL (squares), and 4 mg/mL (diamonds). Values shown represent the mean ± standard deviation of three independent experiments.

References: Micsonai et al. Nucleic Acids Res. 46:W315-22 (2018), Micsonai et al. PNAS 11:E3095-103 (2015)

PDB ID: **2Q8X** Entire structure Length: **663**

Secondary structure composition

DSSP [a]	%
3-10-helix	**2.1**
Alpha helix	**37.6**
Pi helix	**0.0**
Turn	**12.8**
Strand	**16.0**
Bridge	**2.7**
Bend	**11.2**
Loop + missing	**17.6**

BeStSel	%
Helix 1	**23.1**
Helix 2	**14.5**
Antiparallel 1	**0.0**
Antiparallel 2	**0.0**
Antiparallel 3	**0.0**
Parallel	**16.0**
Turn	**12.8**
Others	**33.6**

SELCON [b]	%
Helix 1	**23.1**
Helix 2	**16.6**
Strand 1	**10.6**
Strand 2	**5.4**
Turn	**21.7**
Others	**22.6**

CATH Info [c]

CATH info is provided on the basis of chains. Please, select chains below.

Further references:
[a] : Dictionary of protein secondary structure: pattern recognition of hydrogen-bonded and geometrical features.
Kabsch W., Sander C.
Biopolymers. 1983 Dec; 22(12):2577-2637. PMID: 6667333
[b] : Estimation of the number of alpha-helical and beta-strand segments in proteins using circular dichroism spectroscopy.
Sreerama N, Venyaminov SY, Woody RW.
Protein Sci. 1999 Feb;8(2):370-380. PMID: 10048330
[c] : CATH: A Hierarchic Classification of Protein Domain Structures.
Orengo CA, Michie AD, Jones S, Jones DT, Swindells MB, Thornton JM.
Structure. 1997 Aug 15;5(8):1093-108. PMID: 9309224

Figure S5. Secondary structure composition of the crystalline structure of XT6 (PDB 2Q8X) analyzed using the BeStSel web server.
